# H-rev107 Regulates Cytochrome P450 Reductase Activity and Increases Lipid Accumulation

**DOI:** 10.1371/journal.pone.0138586

**Published:** 2015-09-18

**Authors:** Fu-Ming Tsai, Mao-Liang Chen, Lu-Kai Wang, Ming-Cheng Lee

**Affiliations:** 1 Department of Research, Taipei Tzuchi Hospital, The Buddhist Tzuchi Medical Foundation, New Taipei City, Taiwan; 2 Department of Internal Medicine, College of Medicine, National Taiwan University, Taipei, Taiwan; Institute of Biochemistry and Biotechnology, TAIWAN

## Abstract

H-rev107 is a member of the HREV107 type II tumor suppressor gene family and acts as a phospholipase to catalyze the release of fatty acids from glycerophospholipid. H-rev107 has been shown to play an important role in fat metabolism in adipocytes through the PGE2/cAMP pathway, but the detailed molecular mechanism underlying H-rev107-mediated lipid degradation has not been studied. In this study, the interaction between H-rev107 and cytochrome P450 reductase (POR), which is involved in hepatic lipid content regulation, was determined by yeast two-hybrid screen and confirmed by using *in vitro* pull down assays and immunofluorescent staining. The expression of POR in H-rev107-expressing cells enhanced the H-rev107-mediated release of arachidonic acid. However, H-rev107 inhibited POR activity and relieved POR-mediated decreased triglyceride content in HtTA and HeLa cervical cells. The inhibitory effect of H-rev107 will be abolished when POR-expressing cells transfected with PLA_2_-lacking pH-rev107 or treated with PLA_2_ inhibitor. Silencing of H-rev107 using siRNA resulted in increased glycerol production and reversion of free fatty acid-mediated growth suppression in Huh7 hepatic cells. In summary, our results revealed that H-rev107 is also involved in lipid accumulation in liver cells through the POR pathway via its PLA_2_ activity.

## Introduction

H-rev107 was identified in H-RAS-resistant murine fibroblasts [1] and has also been identified as HRASLS3 [2] and PLA2G16 [3]. H-rev107 is encoded by a 3.5-kb mRNAthat can be translated to a 17.9-kDa protein that belongs to the HREV107 protein family, which includes HREV107 [4], retinoid-inducible gene 1 (RIG1) [5], HRASLS2 [6], HRLP5 [7], and HRASLS [8]. The proteins in this family contain a proline-rich motif located at the N-terminus followed by a conserved H-box, an NC domain and a hydrophobic membrane-anchoring domain at the C-terminus [9, 10]. HREV107 family proteins play important roles in the regulation of cellular growth, differentiation, and apoptosis [11–17].

Several recent studies demonstrated that each member of the HREV107 protein family might act as a phospholipase/acyltransferase to catalyze the release of fatty acids from glycerophospholipid or the transfer of an acyl group from glycerophospholipid to the hydroxyl group of lysophospholipid [7, 18–21]. H-rev107 null mice have a markedly higher rate of lipolysis and have drastically reduced adipose tissue mass and triglyceride content with normal adipogenesis. Ablation of H-rev107 prevents obesity from high fat feeding or leptin deficiency in an obese mouse model [22]. This study suggests that H-rev107 plays an important role in fat metabolism, which may contribute to the regulation of cellular differentiation in both normal and cancer cells.

Structure and function data from HREV107 family proteins show that C-terminal transmembrane domains target the protein to endomembranes for critical functions [6, 12, 13, 16, 23–25]. The N-terminal 124 amino acid region is required for RIG1- dependent keratinocyte differentiation [24]. The proline-rich region binds to protein phosphatase 2A and inhibits the enzyme's activity [2]. Both the proline-rich region and the C-terminal transmembrane domain play important roles in H-rev107-mediated down-regulation of peroxisomes through binding to Pex19 and inhibiting its chaperone activity [26]. The NC domain, specifically at the Asn-112Cys-113 motif, is important for RIG1 function during cell death [12]. His-154 and Cys-241 play crucial roles in the phospholipase A_1_/A_2_ activity of rat HRLP5 [7].

Cytochrome P450 reductase (POR), also known as NADPH:ferrihemoprotein oxidoreductase, is a endoplasmic reticulum membrane-bound enzyme required for electron transfer from NADPH to cytochrome P450 [27]. Cytochrome P450s play important roles in the metabolism of steroids, cholesterol, bile acid, carcinogens and drugs [28–31]. POR is required for the activity of all microsomal P450 enzymes. Disruption of POR in mice results in embryonic lethality [32]. Hepatic POR-null mice develop a severe hepatic lipidosis and an altered fatty acid profile [33, 34]. Several missense mutations (R457H, V492E, C569Y, and V608F) in the POR genes have been found in patients with deficient activities of multiple steroidogenic enzymes and with and without Antley-Bixler syndrome [35].

We found that H-rev107 interacted with POR using yeast two-hybrid screening using the NC domain of H-rev107 as bait. H-rev107 is considered a phospholipid-metabolizing enzyme that releases the free fatty acids and lysophospholipids from phosphatidylcholine [3, 19]. Given that POR is involved in the accumulation of triglycerides in hepatocytes, we hypothesized that H-rev107 acts as a regulator for lipid accumulation in cells. In this study, we demonstrated a role for H-rev107 in lipid accumulation in liver cells through the POR pathway via its PLA_2_ activity.

## Materials and Methods

### Yeast two-hybrid screening

The yeast two hybrid system including yeast strain CG-1945 and Y187, the pAS2-1 vector and a cDNA library derived from HeLa cervical cancer cells was a generous gift of Dr. T.-C Tsai (Department of Microbiology, Immunology and Biopharmaceuticals, National Chiayi University, Chiayi, Taiwan). To generate expression vector for DNA-binding domain of the transcriptional activator GAL4 (GAL4 BD)-tagged NC domain of the H-rev107, truncated H-rev107 cDNA fragments were amplified from pH-rev107-myc [36] using 5' (5'- CCGAATTCAGCATCATGTCTGCTTTGAC-3') and 3' (5'- ACGGATCCTCAATCTCTGACCTGATCACTCC -3') primers and then subcloned in-frame into the pAS2-1 to generate pAS2-1-H-rev107_49–132_. The plasmid pAS2-1-H-rev107_49–132_ was used as bait in screening of the HeLa cells cDNA library that was fused to the GAL4 activation domain (AD) vector pACT2. The GAL4 BD/H-rev107_49–132_ and the AD/HeLa cells cDNA library hybrids were co-transformed into yeast strain CG-1945 (Trp^-^, Leu^-^) for library screening. Histidine-positive (His^+^) colonies were then assayed for β-galactosidase activity. Plasmids from putative His^+^LacZ^+^ positive clones were isolated from the yeast and then individually transformed into *E*. *coli* HB101 cells for amplification. These cDNA-containing Y187 yeasts were then mated with CG-1945 that contains GAL4 BD/H-rev107_49–132_. The resulting diploids were plated on selection medium without tryptophan, leucine and histidine and the β-galactosidase activity was determined. Clones containing cDNA for possible H-rev107-binding proteins were screened by PCR. The amplified products were analyzed by using 1% agarose gel to estimate the size.

### Construction of expression vectors

A plasmid encoding the GFP-tagged mouse P450 (cytochrome) oxidoreductase (POR) was purchased from Origene Technologies (Rockville, MD, USA). To generate pPOR-Flag, the POR cDNA fragment was amplified from the pGFP-POR using 5' (5'-CAAAGCTTCGATGGGGGACTCTCACGAAGAC-3') and 3' (5'-TCGGATCCGGCTCCATACATCCAGCGAGTAG-3') primers and then subcloned in-frame into *Hin*dIII-*Bam*HI sites of the pPCR3.1-Flag. To generate expression vectors for GST-tagged H-rev107 variants, full-length and truncated H-rev107 cDNA fragments were amplified from pH-rev107-myc using 5' (5'- GGGATCCGATGCTAGCACCCATACC-3') and the respective 3’ primers for full-length H-rev107 (5'-GCAAGCTTTTGCTTCTGTTTCTTGTTTCTGGAGAG-3'), H-rev107_1–132_ (5'-GCAAGCTTATCTCTGACCTGATCACTCCG-3'), and H-rev107_1–48_ (5'-GCAAGCTTGGCTGCCCCAGCTCCTG-3'). To generate expression vectors for GST-tagged POR variants, full-length and truncated POR cDNA fragments were amplified from pPOR-Flag using 5' (5'-CGGGATCCGATGGGGGACTCTCACGAAG-3') and the respective 3’ primers for full-length POR (5'-GCAAGCTTGCTCCATACATCCAGCGAGTAGCG-3'), POR_1–603_ (5'-GCAAGCTTGACCTTGTGGGCCTGCTCAC-3') and POR_1–430_ (5'-GCAAGCTTAATGGCTAGGATGTGCCTCCGG-3'). The amplified cDNA described above were then digested with *Bam*HI and *Hin*dIII and inserted into the pET41b vector (Novagen, EMD Bioscience, Cambridge, MA, USA). Expression vectors (pH-rev107 H23L and pH-rev107 C113S) with H-rev107 point mutants containing the myc epitope were generated by site-directed mutagenesis using a single primer as described previously [37]. Briefly, H-rev107 H23L and C113S plasmids were constructed by amplification of the pH-rev107-myc plasmid using primers for H-rev107 H23L (5'-CCGCCCTATGTACAGACTCTGGGCCATCTATGTTGGTGATGG-3') and H-rev107 C113S (5'-GCTGACCAGCGAGAACTCTGAGCACTTTGTGAATGAACTACG-3'). To generate Flag-tagged POR mutated expression vectors, mutated POR plasmids were generated via site-directed mutagenesis by amplification of the pPOR-Flag using primers containing mutations at amino acids R454H (5'-CCCGAGGCTGCAGGCCCACTACTATTCCATTGCCTCGTCGTC-3'), V489E (5'-GACGAGTGAACAAGGGGGAGGCCACCAGCTGGCTTCGGACCAAG-3'), C566Y (5'-CGCTGCTCTACTACGGCTACCGGCGCTCGGATGAGGACTATCTG-3'), and V603F (5'-GTGAGCAGGCCCACAAGTTCTATGTTCAGCACCTGCTCAAGAG-3'). cDNA sequences for H-rev107 and POR and the expression of fusion proteins were confirmed by DNA sequencing and western blotting, respectively.

### Cell culture and transfection

HeLa and HeLa Tet-off (HtTA) cervical cancer cells were obtained from Dr. T.-C. Chang (Department of Biochemistry, National Defense Medical Center, Taiwan) and maintained in RPMI-1640 medium supplemented with 25 mM HEPES, 26 mM NaHCO_3_, 2 mM _L_-glutamine, penicillin (100 units/mL), streptomycin (100 μg/mL), and 10% fetal bovine serum (FBS) at 37°C in 5% CO_2_. The hepatoma derived cell line Huh7 was obtained from Dr. C.-S. Hsu (Department of Internal Medicine, Taipei Tzuchi Hospital, The Buddhist Tzuchi Medical Foundation, Taiwan). Cells were grown in DMEM medium supplemented with 10% FBS, 2 mM _L_-Glutamine, 100 units/mL penicillin and 10 μg/mL streptomycin at 37°C under 5% CO_2_, in a 95% humidified atmosphere. Cells plated in culture dishes were transfected with the expression vectors using liposome mediated-transfection. Briefly, plasmids and lipofectamine 2000 (Invitrogen, Carlsbad, CA, USA) were diluted in Opti-MEM medium and then mixed with plasmids at room temperature for 15 min. The DNA-lipofectamine 2000 complexes were then added to cells for 5 h at 37°C. Cells were refreshed with complete medium for 24 h at 37°C for further analysis. To establish stable cells expressing empty vector or POR-Flag, HeLa cells were transfected with pPCR3.1-Flag or POR-Flag plasmid and selected by medium containing 400 μg/ml G418 (Invitrogen) for 30 days.

### Immunoprecipitation and western blotting

Cells were lysed in modified RIPA buffer (20 mM Tris-HCl [pH 7.5], 100 mM NaCl, 1% NP-40, 30 mM sodium pyrophosphate) containing a protease inhibitor cocktail (Roche Diagnostics, Mannheim, Germany) and phosphatase inhibitors. Cell lysates containing 500 μg of protein were first incubated for 2 h at 4°C with 3.2 μg of anti-MYC (Invitrogen) or anti-FLAG (Sigma, St. Louis, MO, USA) monoclonal antibody and then incubated for 2 h at 4°C with 20 μL of protein G plus/protein A agarose (Calbiochem, Cambridge, MA, USA). Alternatively, cell lysates containing 5 mg of protein were incubated first with 4 μg of normal rabbit IgG (Santa Cruz Biotechnology, Santa Cruz, CA), anti- H-rev107 (Abnova, Taipei, Taiwan), or anti-POR (Santa Cruz Biotechnology) antibody for 12 h at 4°C and then incubated with 20 μL of protein G plus/protein A agarose (Calbiochem) at 4°C for 2 h. Immunoprecipitated complexes were analyzed by western blotting using an anti-FLAG or anti-MYC antibody after washing three times with PBS. For western blotting, 20 μg of protein were separated on 15% polyacrylamide gels and transferred to polyvinylidene fluoride membranes. After blocking, membranes were incubated for 12 h at 4°C with anti-MYC, anti-FLAG, or anti-Actin (Sigma) antibody and then incubated with horseradish peroxidase-conjugated goat anti-mouse antibody at room temperature for 1 h. An ECL kit (Amersham, Bucks, UK) was used to detect the substrate reaction.

### Confocal and immunofluorescent analysis

HtTA cells (1×10^5^) were plated on poly-L-lysine-coated coverslips in 35-mm dishes in growth medium. Cells were then transfected with 500 ng of H-rev107 along with 500 ng pGFP-POR expression vector for 18 h. The cells were washed and fixed with 4% paraformaldehyde and then incubated with anti-MYC antibody followed by Alexa fluor 633 anti-mouse IgG antibody (Invitrogen). The cells were then analyzed for H-rev107 and GFP-POR expression with a Leica TCS SP5 scanner (Leica, Bensheim, Germany).

### Protein expression and *in vitro* pull-down assay

Both GST fusion with full length and truncated H-rev107 and POR were isolated and purified from *E*. *coli* over-expressing the respective proteins. We expressed the inserted gene via induction of T7 polymerase with 1 mM isopropyl β-D-1-thiogalactopyranoside in BL21(DE3) *E*. *coli* after cells reached mid-log phase. Purification of GST fusion proteins was facilitated by glutathione-Sepharose resin (Amersham Biosciences). Purified proteins were confirmed by western blot analysis using antibodies against polyhistidine (Sigma) (data not shown). For *in vitro* pull-down assay, H-rev107-myc or POR-Flag were *in vitro* transcribed and translated in TNT^®^ Quick Coupled Transcription/Translation Systems (Promega Corporation, Madison, WI, USA) according to the manufacturer’s instruction. *In vitro* transcribed and translated H-rev107-MYC were incubated with 10 μg GST-POR deletions, whereas *in vitro* transcribed and translated POR-FLAG interacted with GST-H-rev107 deletion variants at 4°C for 2 h with *in vitro* interaction buffer (50 mM HEPES, pH 7.4, 150 mM NaCl, 1 mM EDTA, 1 mM EGTA, 10% (v/v) glycerol, 1% Triton X-100, and a mix of protease inhibitors), after washing three times with interaction buffer and twice with PBS, bound proteins were eluted with 1×SDS sample buffer, separated by denaturing SDS-PAGE, and analyzed with anti-FLAG or anti-MYC antibodies.

### *In Vitro* Phospholipase A_2_ (PLA_2_) activity assay

The PLA_2_ activity assay was performed as described previously [3]. Briefly, the reaction (1 ml) at 25°C contained the following components: 100 μM 1-palmitoyl-2-linoleoyl-PC (Sigma), 0.36 μg/ml lipoxygenase (Sigma), and 2 mM CaCl_2_ in 50 mM Tris buffer (pH 8.5) containing 2 mM deoxycholate. The reaction was started by adding recombinant H-rev107 protein [36] to the reaction buffer and PLA_2_ activity was recorded with a DU530 UV/VIS spectrophotometer (Beckman coulter Inc, Brea, CA, USA) by measuring the increase in absorbance at 234 nm due to the formation of the hydroperoxide (ε234 = 25,000 M^-1^ cm^-1^) in a kinetic mode.

### Arachidonic acid (AA) release assay

Cells were cultured onto 6-well plates overnight and then transfected with 500 ng of pH-rev107-myc along with various (0.5–1 μg) concentrations of the POR-Flag expression vector in complete medium for 12 h. Cells were washed and then incubated in RPMI medium containing 1 mg/mL BSA for 6 h. The levels of AA in the supernatants were determined using a human AA ELISA kit (Cusabio Biotech, Wuhan, Hubei, China).

### POR activity assay

Cells plated in 6-well plates were transfected with 500 ng of POR-Flag expression plasmids along with various concentrations of H-rev107-myc (0.5–1 μg) and then incubated for 24 h in complete medium containing 10 μM arachidonyl trifluoromethyl ketone (AACOCF3, Cayman Chemical, Ann Arbor, MI, USA), 5μM methyl arachidonyl fluorophosphate (MAFP, Sigma), or 2 μM Bromoenol lactone (BEL, Sigma). Cells were lysed in modified RIPA buffer. POR activity assay was performed as described previously[38]. Briefly, the reaction was conducted in a 1 mL of 0.3 M potassium phosphate buffer (pH 7.7) containing 10 μL of cell lysates, 40 μM horse heart cytochrome c (Sigma), and 1 mM Potassium cyanide. After 100 μM NADPH (Sigma) was added, the POR activity was recorded with a DU530 UV/VIS spectrophotometer in a kinetic mode.

### Staining of lipid droplets and measurement of glycerol

Cells plated in 6-well plates were transfected with POR-Flag expression plasmids along with H-rev107-myc and then incubated for 24 h in complete medium containing 10 μM AACOCF3, 5 μM MAFP, or 2 μM BEL. Cells were washed and then incubated in 1% BSA/RPMI medium containing 10 mg/mL oleic acid for 3 h. Cells were fixed with 4% paraformaldehyde and then stained with Nile Red (Invitrogen). Alternatively, cells were transfected with the indicated expression vectors for 24 h and incubated with 10 mg/mL oleic acid for 3 h. Cell were washed and then incubated in 1% BSA/RPMI medium for another 24 h. Glycerol levels in the supernatants were determined using a glycerol colorimetric assay kit (Cayman).

### RNA interference

siRNA oligonucleotides were synthesized by Sigma. Two H-rev107 siRNAs targeted to nucleotides 874 to 892 (5’-GGAGUCAUGUUCUCAAGAAtt-3’) and 486 to 504 (5’-CAGACACUGGGCCAUCUAUtt-3’) were synthesized based on Genbank accession NM_007069.3. POR siRNAs targeted to nucleotides 885 to 903 (5’-GGCUGAAGAGCUACGAGAAtt-3’) and 1498 to 1516 (5’-GCACAUCUGUGCGGUGGUUtt-3’) were synthesized according to Genbank accession NM_000941.2. The universal NC control siRNA (Sigma) was used as a negative control.

### Free fatty acids (FFA) treatment and analysis of cell death and cell viability

Huh7 cells were plated in triplicate in 24-well plates at a density of 2×10^4^ cells per well in DMEM medium containing 10% FBS and incubated overnight. The cells were transfected with 40 pmol various H-rev107 siRNA, POR siRNA or universal NC siRNA and then immediately refreshed with complete medium. Twenty-four hours after transfection, cells were incubated in growth medium containing a fresh mixture of FFA (palmitic:oleic acid in molar ratio 1:2), respectively, or ethanol vehicle (0.1%) for another 24 h. Cells were analyzed for viability using theWST-1 reagent (100 μL Roche Diagnostics). The percentage of cell viability relative to ethanol-treated cells expressing NC siRNA was defined as [(A450–A650) of target gene siRNA-transfected cells/(A450–A650) of ethanol-treated cells expressing NC siRNA] ×100%. Measurement of the release of lactate dehydrogenase (LDH) using the Cytotoxicity Detection Kit (Roche Diagnostics) was used to evaluate cell death. Percentage of LDH release was defined as [(A490–A650) of target gene siRNA-transfected cells /(A490–A650) of NC siRNA transfected cells]×100%.

### Cytokine assays

The supernatants of FFA-treated Huh7 cells were collected and used as materials to analyze cytokine production. IL-6, IL-8, and TNF-α were measured in Huh7 cell supernatants by ELISA (Peprotech, Rock Hill, NJ, USA).

### Statistical analysis

Data are represented as the mean±SD of at least triplicate studies. Statistical analyses were performed using one-way ANOVA with Dunnett's post hoc test for comparison of more than two groups. Student’s t test was used for comparisons between two groups. A *p*-value < 0.05 was considered statistically significant.

## Result

### H-rev107 associates and co-localizes with POR

**Fig 1:**
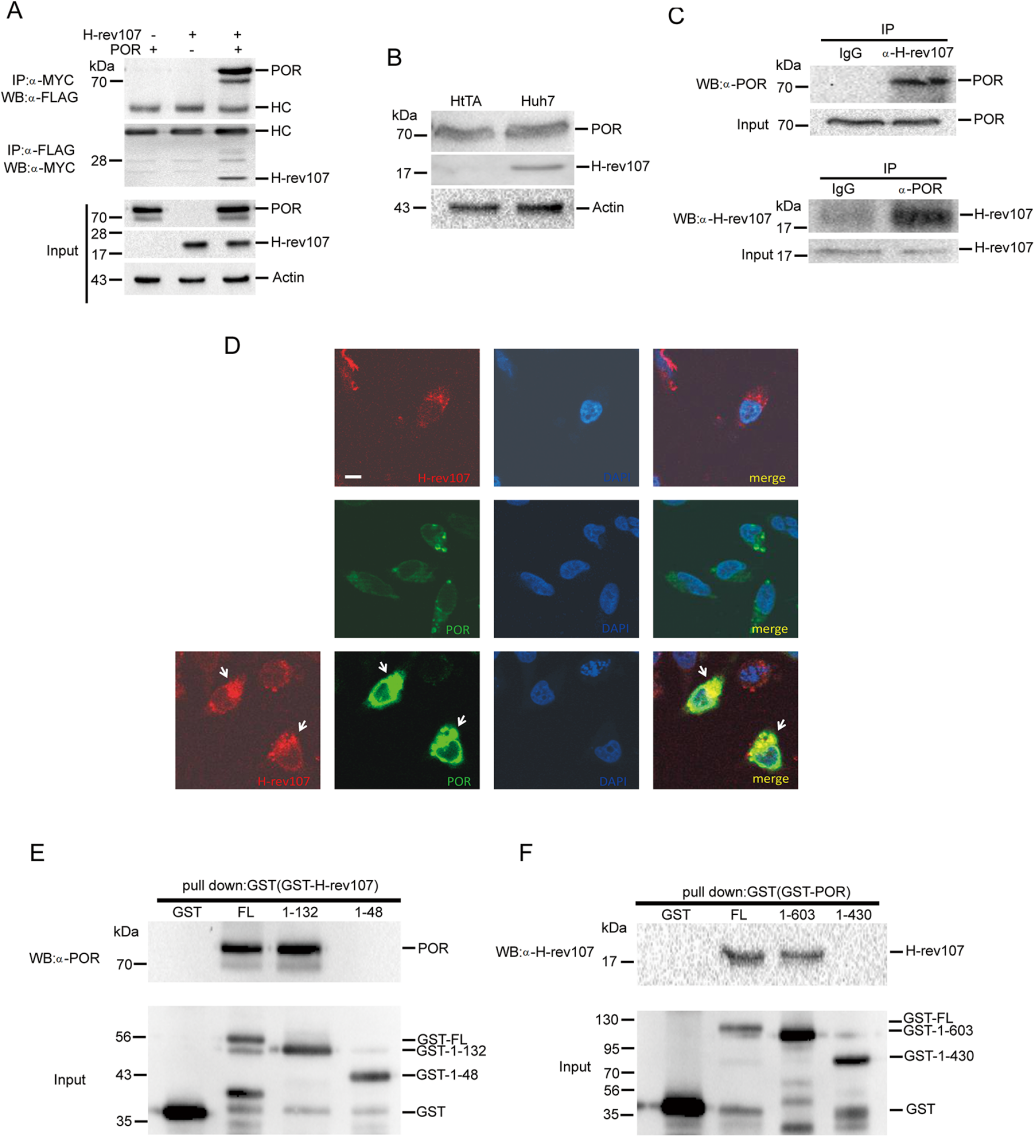
H-rev107 associates and co-localizes with POR. HtTA cells plated in a 10-cm dish were transfected with H-rev107-myc and POR-Flag expression vector for 24 h. The cell lysates were prepared, and the interaction between H-rev107 and POR was analyzed by immunoprecipitation followed by western blot analysis. Immunoprecipitates were resolved by SDS-PAGE and immunoblotted using the anti-Flag antibody or anti-myc antibody (A). Total cellular extracts from HtTA or Huh7 cells were subjected to western blot analysis for H-rev107, POR, and actin (B). Huh7 cell lysates were prepared, and the interaction between H-rev107 and POR was analyzed by immunoprecipitation using anti-H-rev107- or POR-specific antibodies, respectively, followed by western blot analysis (C). HtTA cells were cotransfected with GFP-POR and H-rev107 expression vectors for 18 h. The cells were fixed and then incubated with anti-myc antibody followed by Alexa fluor 633 anti-mouse IgG antibody. The localization of H-rev107 (red), POR (green), and nuclei (blue) were analyzed using a laser scanning confocal microscope. Yellow fluorescence (arrows) evident in the merged images indicates co-localization of the H-rev107 and POR (D). The GST-tagged fusion proteins of H-rev107 (E) or POR (F) were purified, and the interaction between H-107 and POR was analyzed by GST pull down followed by Western blot analysis. Scale bar: 10 μm.

We used the *in vivo* yeast two-hybrid system to identify possible proteins that interact with H-rev107 in this study. Three different clones exhibiting His^+^LacZ^+^ positive interaction phenotypes were identified. The DNA sequencing result matched that of nucleotides 195 to 1144 in reference ribosomal protein, large, P0 (RPLP0) mRNA (accession number: NM_001002.3), nucleotides 8 to 544 in reference mitochondrial ribosomal protein L21 (MRPL21) mRNA (accession number: NM_181514.1) and nucleotides 1394 to 2509 in reference POR mRNA (accession number: NM_000941.2), respectively. The POR protein was selected and the specificity of the interaction between POR and H-rev107 was further verified. To confirm the interaction between H-rev107 and POR, we first constructed expression vectors that synthesized recombinant proteins containing Flag-tagged POR. Interaction was confirmed in HtTA cervical cancer cells. H-rev107 protein immunoblots from POR co-transfected immunoprecipitates indicates the presence of H-rev107 in the POR pull-down protein complexes prepared *in vitro* (Fig 1A). Similarly, POR was present in the H-rev107 immunoprecipitates (Fig 1A). The endogenous expression of H-rev107 and POR was confirmed by western blotting in cells derived from HtTA cells and Huh7 hepatoma cells. The expression of POR was detected in both HtTA and Huh7 cell lysates, whereas H-rev107 was only detected in cell extract prepared from Huh7 cells (Fig 1B). The endogenous H-rev107-POR interaction was confirmed by reciprocal co-immunoprecipitation in the Huh7 cell line (Fig 1C). To further examine the distribution of H-rev107 and POR expression in cells, we first analyzed the subcellular localization of these proteins. HtTA cells were transfected with pGFP-POR or p-H-rev107-myc expression vector for 18 h and then stained with ER or Golgi specific markers. Both H-rev107 and POR primarily co-localized with ER specific PDI protein and to lesser extent with Golgi specific GM130 protein, suggesting that both H-rev107 and POR primarily localize to the ER areas within the cells (S1 Fig). Additionally, the results show that both GFP-POR fusion protein and H-rev107 were primarily distributed in the perinuclear region, especially at the ER (S2 Fig) and that most of the H-rev107 and GFP-POR proteins were co-localized (yellow) in co-transfected cells (Fig 1D).

The H-REV107 protein family participates in multiple biological functions. Both the membrane-anchoring domain and NC domain are indispensable for this activity [11, 12, 23, 25]. To determine the POR binding domain of H-rev107, we constructed GST-tagged membrane spanning domain deleted H-rev107_1–132_ and both membrane-anchor and NC domain deleted H-rev107_1–48_. The POR protein appeared on immunoblots from GST-H-rev107 and GST-H-rev107_1–132_, but not from GST-H-rev107_1–48_, thus indicating the presence of POR in the NC domain-containing H-rev107 pull-down protein complexes that were prepared *in vitro* (Fig 1E). We further examined the interaction domain of POR with H-rev107 with synthesized recombinant proteins containing GST-tagged POR deletion variants. H-rev107 proteins were immunoprecipitated with GST-POR and GST-POR_1–603_ but not with GST-POR_1–430_ (Fig 1F). The results suggest that a candidate region (from amino acid 430 to 603) in POR protein is necessary for the binding of H-rev107.

### POR enhances H-rev107-mediated AA release

**Fig 2:**
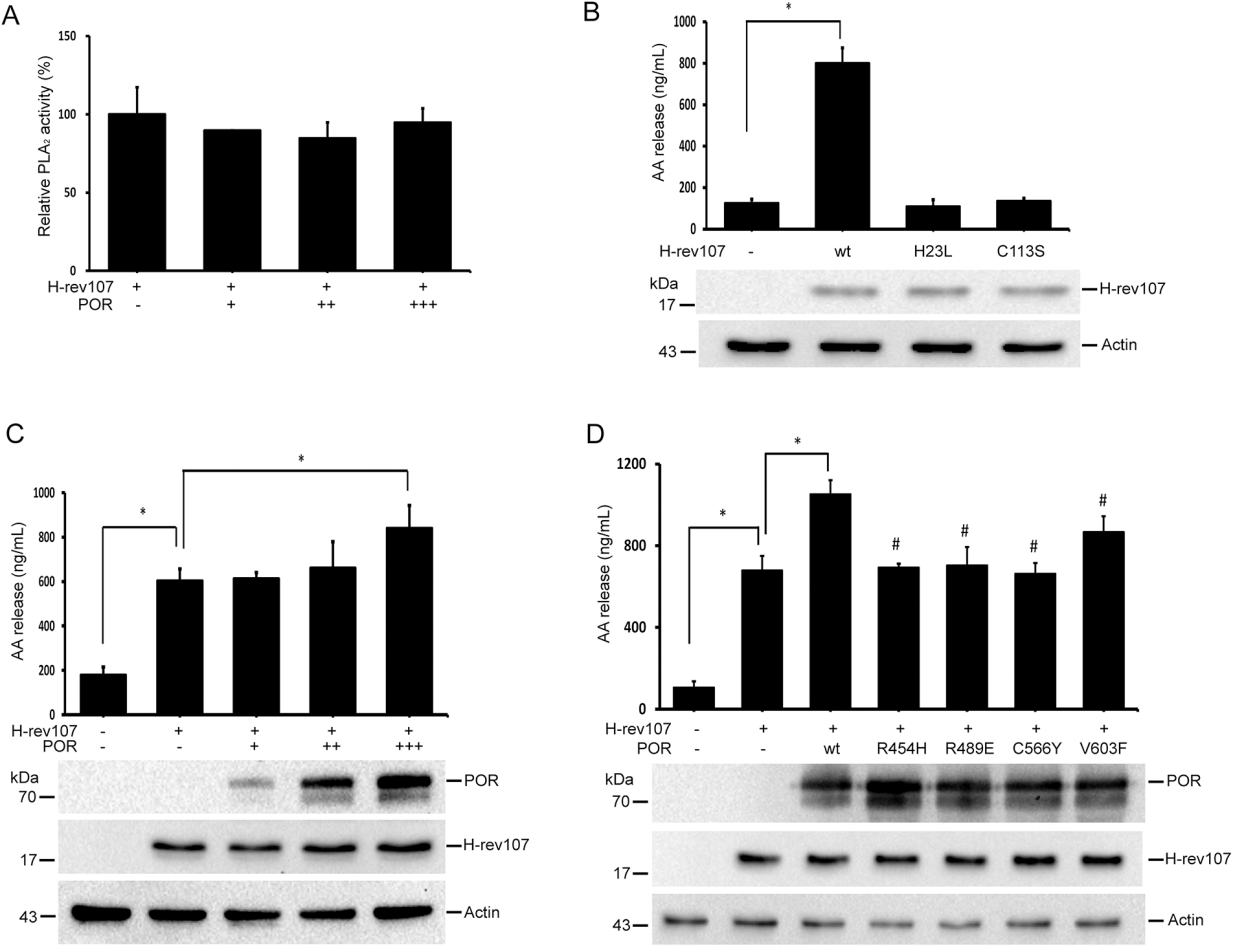
POR enhances H-rev107-mediated AA release. Recombinant H-rev107 (500 ng) was assayed in 50 mM Tris buffer along with 500 ng-1.5 μg of recombinant GST-POR or not for 30 min, and PLA_2_ activity was performed as described in Materials and methods (A). HtTA cells were plated in triplicate into 6-well plates, incubated overnight, and then transfected with the indicated H-rev107 plasmid or control vector for 24 h (B). Cells were transfected with H-rev107 expression or control vector along with 0.5–1 μg of the POR-Flag plasmid (C), or indicated POR-Flag expression vector (D) for 24 h. Cells were serum-starved for 6 h and levels of AA were determined using enzyme immunoassays. Representative results of three independent experiments are shown. *Indicates *p* value <0.05. ^#^Indicates *p* value <0.05 when cells transfected with wild type POR compared to cells transfected with indicated POR plasmid in H-rev107-expressing cells.

H-rev107 is considered a phospholipase A_2_, which may release free fatty acids and lysophospholipid from phosphatidylcholine [3]. Because POR can interact with H-rev107, we next examined the effect of POR on the PLA_2_ activity of H-rev107 *in vitro*. Our result revealed that POR had no effect on the PLA_2_ activity of H-rev107 (Fig 2A). The PLA_2_ activity of H-rev107 in cells, which was assessed as the AA releasing capacity of PLA_2_ in cells, was also evaluated. We found that 24 h expression of wild type H-rev107 a significant increase of 6.1-fold in AA release (Fig 2B). His-154 and Cys-241 of HRLP5 are critical for the PLA_1_/A_2_ activity of HRLP5 [7]. To further examine the role of the corresponding residues His-23 and Cys-113 in H-rev107-mediated AA release in HtTA cells, we constructed constitutive myc-tagged H-rev107 expression vectors that synthesized wild type H-rev107 with single (H23L or C113S) amino acid substitution. We observed no significant AA release in HtTA cells expressing His23 or Cys113 substituted H-rev107 (Fig 2B), indicating both His23 and Cys113 are critical for the PLA_2_ activity of H-rev107.

We further examined the effect of POR on PLA_2_ activity of H-rev107. HtTA cells were co-transfected with the pH-rev107-myc expression vector and the pPOR-Flag or empty control vector. We found expression of H-rev107 increased AA release in HtTA cells by 7.13-fold. In contrast to the *in vitro* result, AA release significantly increased by 9.64-fold in HtTA cells co-transfected with pH-rev107 and highest dose of pPOR-Flag (1 μg) (Fig 2C). Expression of POR only in HtTA cells had no effect on the AA release (data not shown). Several missense mutations in the POR genes have been found in patients with deficient activities in multiple steroidogenic enzymes [35]. We also examined the importance of the corresponding residues Arg454, Arg489, Cys566, and Val603 of POR in H-rev107-mediated AA release in HtTA cells. The H-rev107-induced AA release was elevated in cells expressing POR. Mutations at Arg454, Arg489, or Cys566 of POR did not enhance H-rev107-mediated AA release. Substitution at V603 of POR exerted a mild enhancement on H-rev107-mediated AA release, which increased by 1.28-fold when compared with H-rev107 (Fig 2D). These results indicate that catalytic activity of POR is important for H-rev107-mediated AA release.

### H-rev107 inhibits POR activity

**Fig 3:**
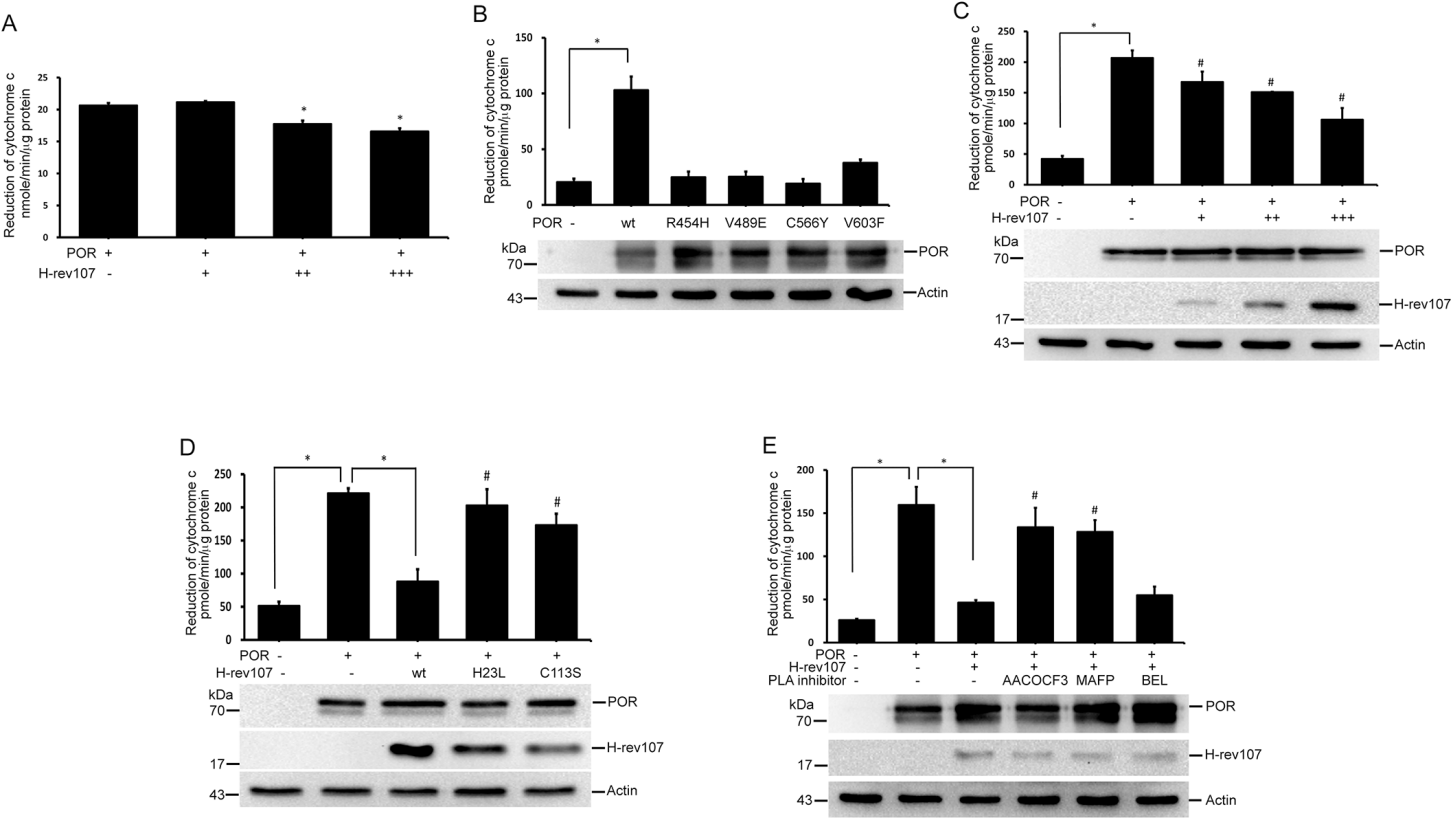
H-rev107 inhibits POR activity. Recombinant GST-POR (500 ng) was assayed in 50 mM Tris buffer along with or without 500 ng-1.5 μg of recombinant H-rev107 for 30 min (A). HtTA cells were plated in triplicate into 6-well plates, incubated overnight, and then transfected with the indicated POR plasmid or control vector for 24 h (B). Cells were transfected with POR expression or control vectors along with 0.5–1 μg of the H-rev107 plasmid (C) or indicated H-rev107-myc expression vector (D) or H-rev107 plasmid in the presence of PLA_2_ inhibitor or DMSO vehicle (D) for 24 h. Cell lysates were prepared, and POR activity was performed as described in Materials and methods. Representative results of three independent experiments are shown. *Indicates *p* value <0.05. ^#^Indicates *p* value <0.05 when cells co-transfected with POR and indicated H-rev107 (C and D) plasmid or treated with PLA_2_ inhibitor (E) compared with cells co-transfected with POR along with empty vector (B) or wild type H-rev107 plasmid (D and E).

We also assessed the effect of H-rev107 on POR activity *in vitro*, which measured the ability for receiving electrons from NADPH and donating them to cytochrome c. Inhibition of POR activity was observed when recombinant GST-POR was co-incubated with a high dose of H-rev107 with maximal 19.7% inhibition in GST-POR protein co-incubated with 1.5 μg recombinant H-rev107 (Fig 3A). We next examined the activities of the POR mutants in cells and expressed each of the mutated constructs in HtTA cells. Expression of wild type POR for 24 h significantly increased the reduction of cytochrome c by 5-fold. All four of the point mutants of POR markedly reduced the ability of cytochrome c to reduce (Fig 3B). We then evaluated the effect of H-rev107 on POR activity in cells. HtTA cells were co-transfected with the pPOR-Flag expression vector and the pH-rev107-myc or control empty vector. Twenty-four hours after transfection we found increased ability of reduction of cytochrome c in cells expressing POR. We found a dose-dependent inhibition in the ability to reduce cytochrome c in H-rev107-expressed HtTA cells (Fig 3C).

To ascertain the role of PLA_2_ activity on H-rev107-mediated suppression of reduction of cytochrome c, we analyzed two missense mutants of H-rev107 on the reduction of cytochrome c. We found neither H23L nor C113S mutated H-rev107 had any effect on POR-mediated reduction of cytochrome c (Fig 3D), although these mutated H-rev107 still bind to POR (S3 Fig). The result suggests that PLA_2_ activity is important for H-rev107 suppression of POR activity. To elucidate the effect of PLA_2_ activity on H-rev107-mediated POR activity suppression, we also performed a series of studies with commercially available PLA_2_ inhibitors. Expression of H-rev107 resulted in downregulation of the reductase ability of cytochrome c by 86% induced by POR. AACOCF3 and MAFP increased the reduction of cytochrome c by 7.7- and 4.6-fold, respectively, in H-rev107-expressing HtTA cells (Fig 3E). BEL had no effect on reduction of cytochrome c activity in H-rev107-expressing HtTA cells. AACOCF3 and MAFP have been shown to inhibit the activity of cytosolic human phospholipase A2, whereas BEL is a mechanism-based suicide substrate [39–41]. The inhibitory effect of AACOCF3 and MAFP but not BEL on H-rev107 is similar to that observed in previous studies [3, 36].

**Fig 4:**
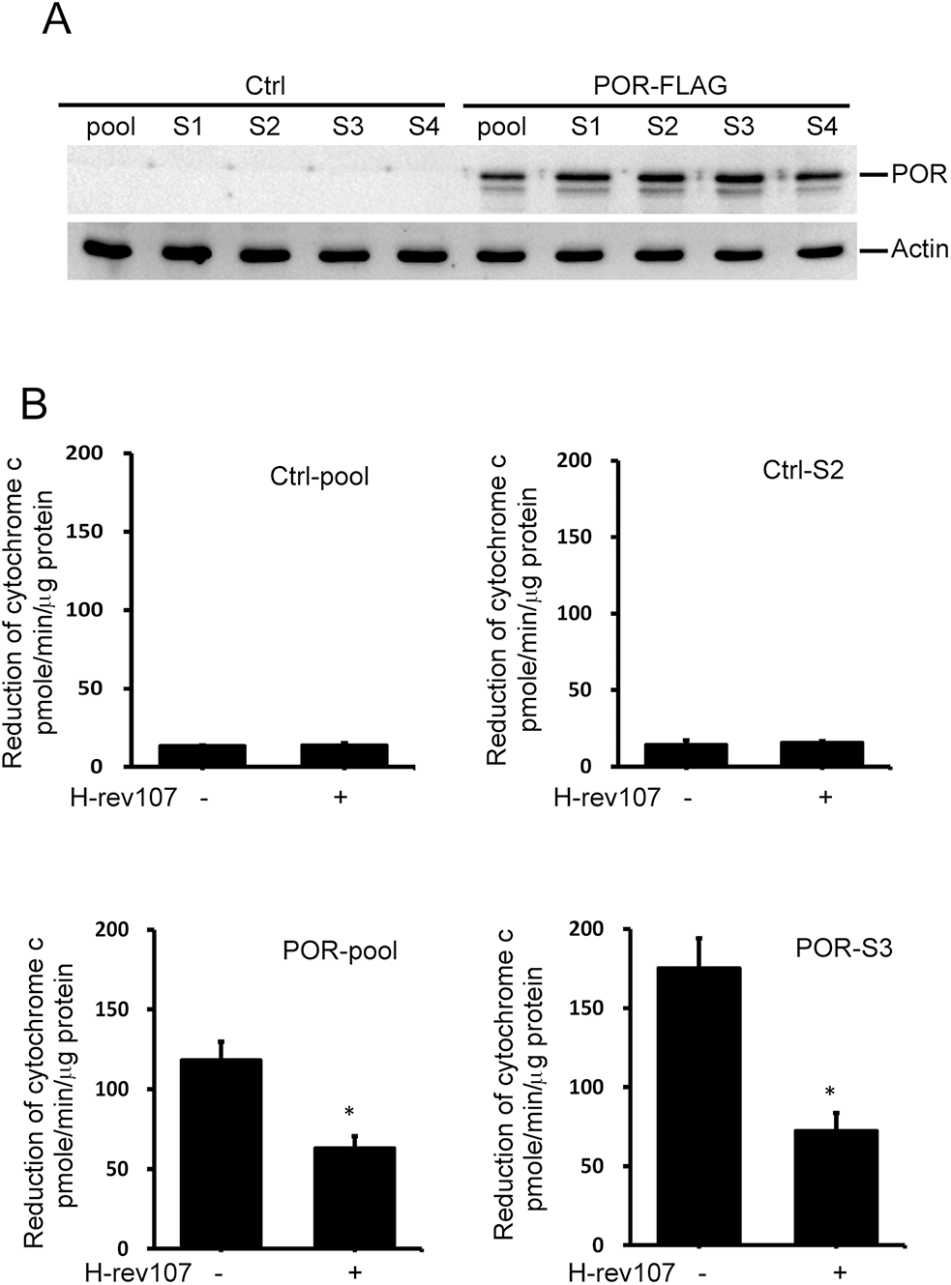
H-rev107 inhibits POR activity in stable HeLa cells. The cell lysates from stable HeLa clones were prepared and expression of POR was determined by western blot analysis (A). Stable HeLa cells were plated in triplicate, incubated overnight, and then transfected with the H-rev107 or empty expression vector for 24 h. Cell lysates were prepared, and POR activity was performed as described in Materials and methods. Representative results of three independent experiments are shown. *Indicates *p* value <0.05.

To further confirm the inhibitory activity of H-rev107 on POR activity, we established stable HeLa cells constitutively expressing POR. We observed constitutive expression of POR in several stable cell lines (Fig 4A). We found that POR activity determined by reduction in cytochrome c increased significantly in POR-expressing stable cells (Fig 4B). Similarly, transiently transfected pH-rev107-myc for 24 h downregulated POR activity in the POR-pool and POR-S3 stable cells by 46.6% and 58.8%, respectively (Fig 4B). H-rev107 had no effect on the POR activity in stable control cells.

### H-rev107 inhibits POR-mediated decreased triglyceride content in cells

**Fig 5:**
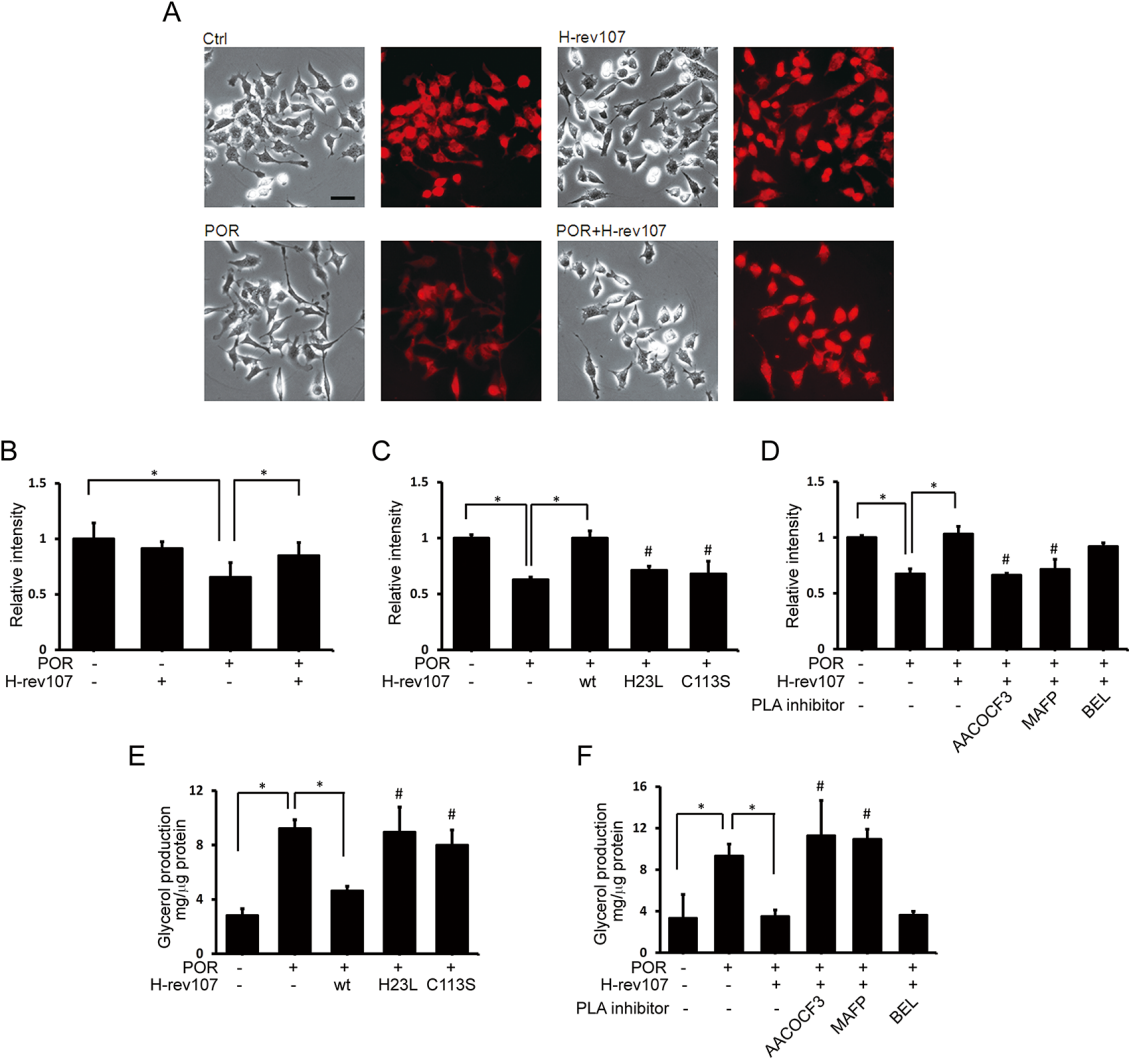
H-rev107 inhibits POR-mediated decreased triglyceride content in cells. HtTA cells were transfected with the indicated expression or control vector in the presence of PLA_2_ inhibitor or DMSO vehicle for 24 h. Cells were washed and then incubated in 1% BSA/RPMI medium containing with oleic acid for 3 h. Lipid droplets were stained with Nile Red (A). The quantitative analysis of Nile Red stained lipid droplet is shown (B-D). After transfection and incubation in medium containing oleic acid, cell were washed and incubated for another 24 h. The glycerol in the media was determined by EIA. Representative results of three independent experiments are shown. *Indicates *p* value <0.05. ^#^Indicates *p* value <0.05 when cells co-transfected with POR and indicated H-rev107 (C and E) plasmid or treated with PLA_2_ inhibitor (D and F) compared to cell co-transfected with POR along with wild type H-rev107 plasmid. Scale bar: 50 μm.

**Fig 6:**
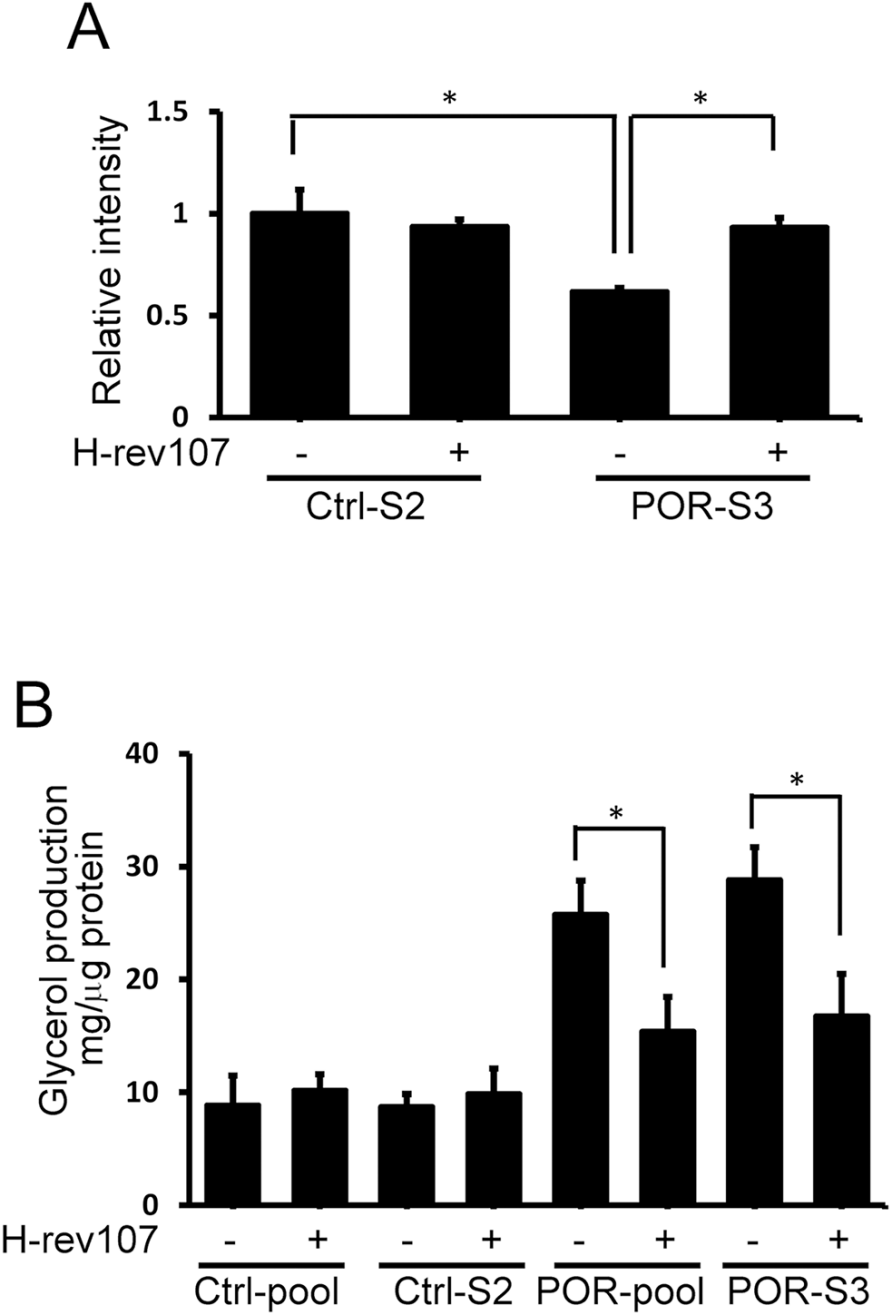
H-rev107 inhibits POR-mediated decreased triglyceride content in stable HeLa cells. Stable HeLa cells were transfected with H-rev107 or empty expression vector for 24 h. Cells were washed and then incubated in 1% BSA/RPMI medium containing oleic acid for 3 h. Lipid droplets were stained with Nile Red and the quantitative analysis of Nile Red stained lipid droplet was shown (A). The glycerol in the media was determined by EIA (B). Representative results of three independent experiments are shown. *Indicates *p* value <0.05.

POR is involved in the accumulation of lipid droplets in the cell [42]. To further understand the role of H-rev107 and POR in cellular triglyceride content, we co-transfected HtTA cells with pH-rev107-myc and pPOR-Flag expression vectors and incubated cells with oleic acid to induce lipid droplet formation. The efficiency of cell transfection with pGFP-POR and pH-rev107 was determined by immunofluorescent analysis. More than 90% of transfected HtTA cells co-expressed H-rev107 and GFP-POR (S4 Fig). HtTA cells expressing POR had fewer lipid droplets in the cytoplasm compared with control or H-rev107-expressing cells (Fig 5A). The increased lipid droplets were observed in H-rev107 and POR co-expressing cells when compared with POR-expressing cell (Fig 5A). Quantification of Nile Red staining showed that cells co-expressing H-rev107 and POR had significantly more Nile Red stain compared with cells expressing POR (Fig 5B). Similarly, over-expressing H-rev107 partially reversed POR-mediated lipid droplet formation suppression in POR-stably expressing HeLa cells (Fig 6A).

We further determined the role of PLA_2_ activity of H-rev107 on POR-mediated lipid droplet formation suppression. We observed less Nile Red stain in POR-expressing cells either co-transfected with H23L or C113S mutated pH-rev107-myc expression vector (Fig 5C) or treated with AACOCF3 or MAFP (Fig 5D) when compared with cells co-transfected with wild type pH-rev107 expression vector. Increased glycerol content in POR-expressing cells indicated the ability of POR on triglyceride degradation (Fig 5E). The glycerol production was decreased in cells co-expressing H-rev107 and POR when compared with cells expressing POR only (Figs 5E and 6B). More glycerol production was observed in POR-expressing cells, either co-expressing H23L or C113S mutated H-rev107 proteins or treated with AACOCF3 or MAFP, compared with cells co-expressing POR and wild type H-rev107 proteins (Fig 5E and 5F).

### Silencing of H-rev107 increases triglyceride degradation

**Fig 7:**
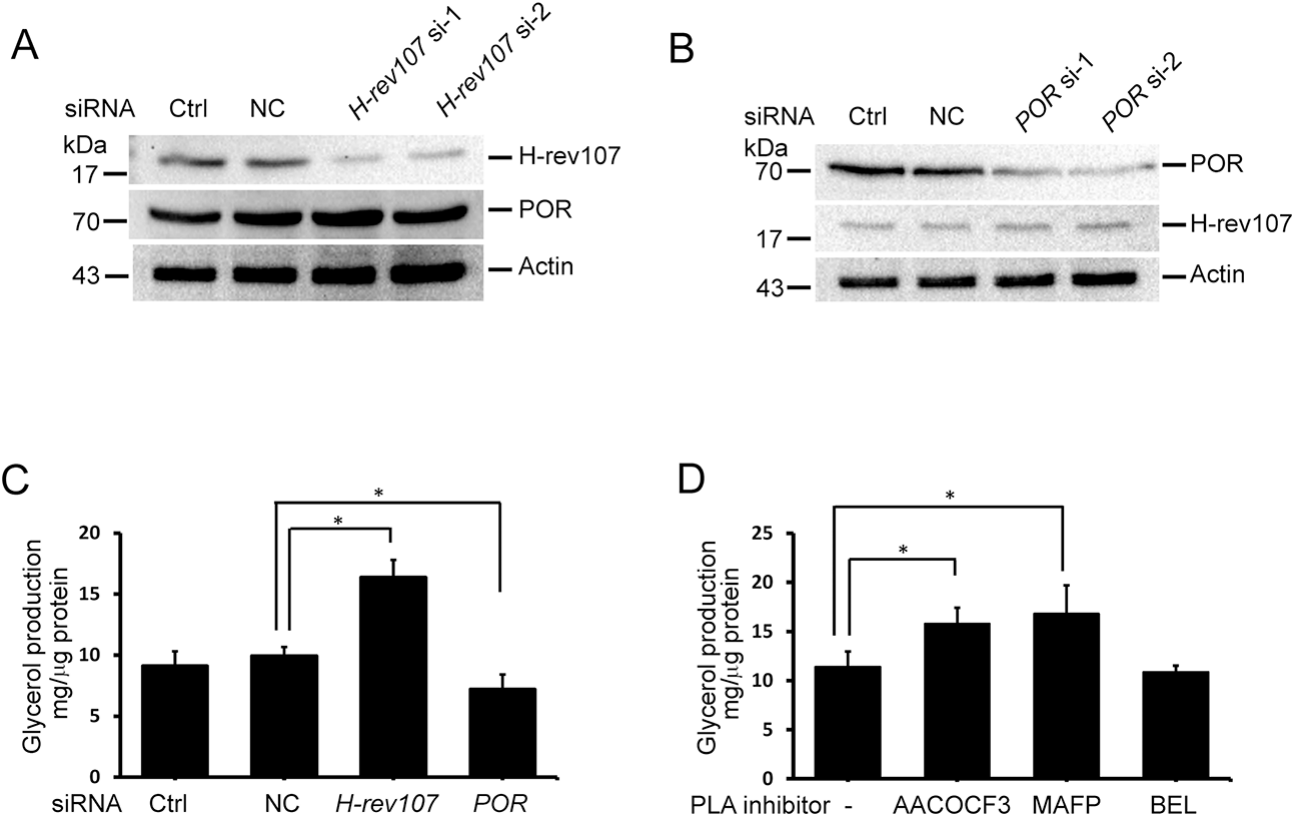
Effects of H-rev107 and POR siRNAs on triglyceride degradation. Huh7 cells were transfected with the indicated siRNA or not for 48 h, cell lysates were prepared, and the expression of H-rev107 (A) or POR (B) was determined using anti-H-rev107 or anti-POR antibodies. Cells were transfected with indicated siRNA (C) or treated with PLA_2_ inhibitor (D) for 24 h and then incubated in 1% BSA/RPMI medium containing with oleic acid for 3 h. Cells were washed and incubated for another 24 h. The glycerol content in the media was determined by EIA. Representative results of three independent experiments are shown. *Indicates *p* value <0.05.

To determine whether H-rev107 contributes to decrease triglyceride content in cells, we examined the effects of H-rev107-silencing on the production of glycerol. Huh7 cells were transfected with each of two different siRNAs to inhibit H-rev107 or POR expression, respectively. As shown in Fig 7A and 7B, the expression of H-rev107 and POR was suppressed in each of two H-rev107 or POR siRNA-transfected Huh7 cells, respectively. The expression of neither H-rev107 nor POR was affected when cells were transfected with universal NC siRNA. Our results showed that glycerol production was markedly increased by 1.65-fold in cells expressing H-rev107 siRNA (Fig 7C). Furthermore, glycerol content was increased by 1.39-fold or 1.48-fold in Huh7 cells treated with AACOCF3 or MAFP, respectively (Fig 7D). BEL had no effect on the glycerol production in Huh7 cells.

### Silencing of H-rev107 inhibits FFA-mediated cell death

**Fig 8:**
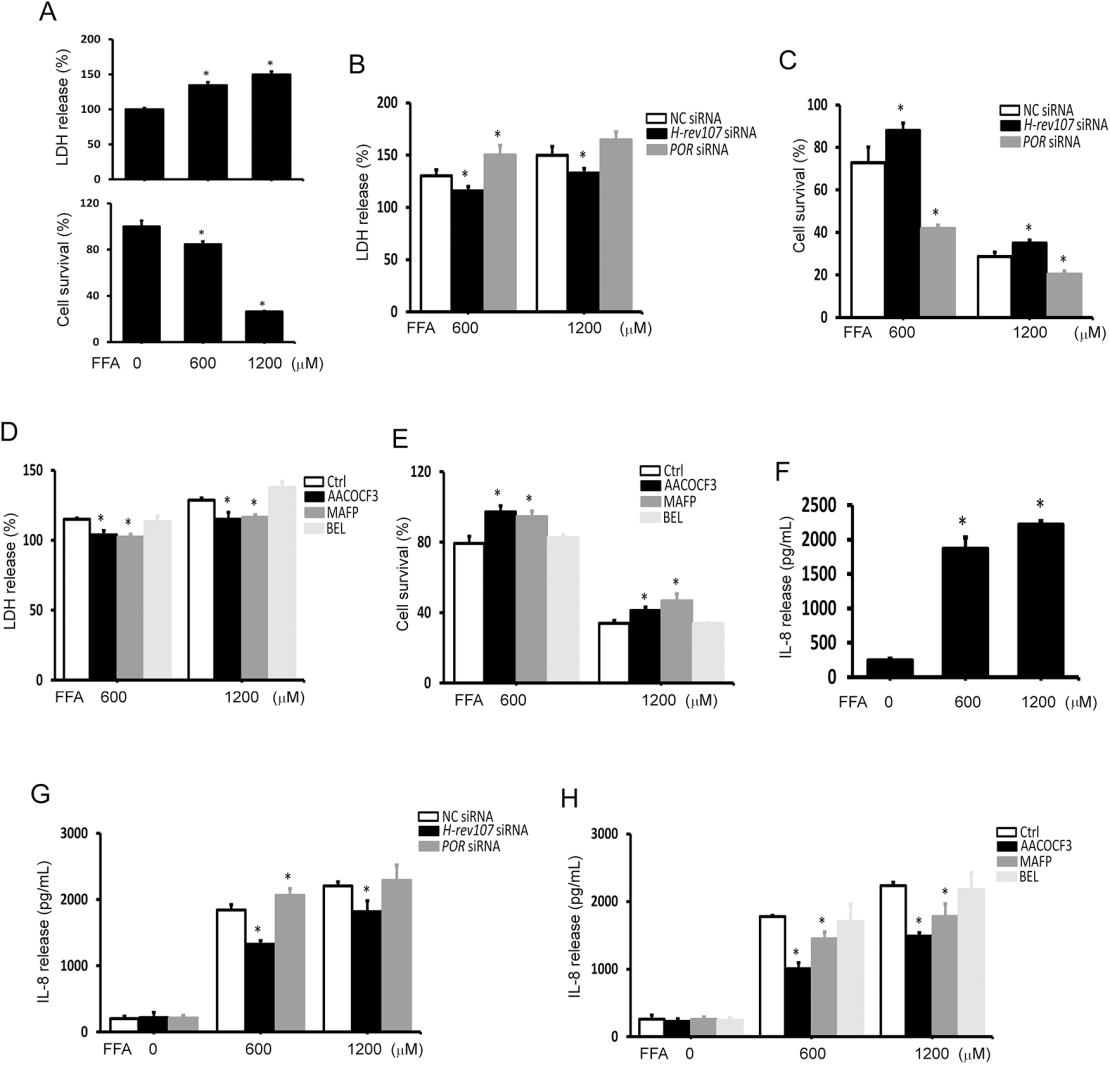
Effects of H-rev107 and POR siRNAs on FFA-mediated suppression of Huh7 cell growth. Huh7 cells were treated with the indicated concentration of FFA for 24 h. Cell death and cell viability was detected by measuring LDH release and with the WST-1 assay, respectively (A). Huh7 cells plated in 24-well plates were transfected with 40 pmol of the indicated siRNA (B and C) or treated with PLA_2_ inhibitor (D and E) for 24 h and then treated with FFA for 24 h. The culture media was collected and cell death was detected by measuring LDH release (B and D). Cell viability was detected with the WST-1 assay (C and E). Huh7 cells were treated with the indicated concentration of FFA for 24 h. The supernatant of cultured Huh7 cells was collected, and the IL-8 release was measured by ELISA (F). Huh7 cells were transfected with the indicated siRNA (G) or treated with PLA_2_ inhibitor (H) for 24 h and then treated with FFA for 24 h. The culture media was then collected and the IL-8 release was measured by ELISA. Representative results of three independent experiments are shown. *Indicates *p* value <0.05 when cells transfected indicated siRNA or treated with PLA_2_ inhibitor compared to NC siRNA expressing cells or vehicle-treated cells.

POR exhibits detoxification activity in liver cells [43]. We further determined the effect of H-rev107 on POR-mediated detoxification activity. Cells treated with toxic amounts of FFA (600 or 1200 μM) for 24 h resulted in significant enhancement of LDH release, ranging from 1.3- to 1.5-fold. Similarly, cell viability determined by the WST-1 assay was significantly reduced by 15.3%-73.5% in cells treated with FFA (Fig 8A). Cell death induced by FFA was inhibited in cells expressing H-rev107 siRNA, as shown by the LDH release assay (Fig 8B). The results of the WST-1 assay demonstrated that cell expressing H-rev107 siRNA alleviated FFA-decreased cell viability (Fig 8C). Similarly, the LDH release and decreased cell viability induced by FFA was inhibited by AACOCF3 and MAFP (Fig 8D and 8E). By contrast, more cell death was observed in cells expressing POR siRNA (Fig 8B and 8C).

The inflammatory cytokines, such as IL-8, has been shown to response FFA stimulation [44]. We observed dose-dependent increases in IL-8 production in FFA-treated Huh7 cells (Fig 8F), similar to the results observed by Chavez-Tapia *et al* [44]. When FFA-treated Huh7 cells were transfected with H-rev107 siRNA, an inhibition of IL-8 production was observed (Fig 8G). The inhibition effect was also observed in FFA-treated Huh7 cells treated with AACOCF3 or MAFP (Fig 8H). However, IL-8 release increased when FFA (600 μM)-treated Huh7 cells was transfected with POR siRNA (Fig 8G). FFA had no effect on the production of IL-6 and TNF-α in Huh7 cells (S5 Fig). Transfection with H-rev107 or POR siRNA did not affect IL-6 or TNF- α (S5 Fig).

## Discussion

H-rev107 is a member of the HREV107 type II tumor suppressor family. Proteins of this family exhibit growth suppression and proapoptotic activities in normal tissues and cancer cells of various origins [6, 12, 13, 16, 45]. The molecular mechanisms underlying the biological functions of HREV107 family proteins have been characterized. Each member of the HREV107 family acts as a phospholipid-related enzyme catalyzing the release of fatty acid from glycerophospholipid [3, 7, 18, 19]. In addition, they have been shown to negatively regulate RAS [1, 6, 11, 17]. PLA_2_ activity plays an important role in H-rev107-mediated RAS suppression [36]. In addition to the PLA_2_ activity of HREV107 protein family, the binding proteins of HREV107 that mediate growth regulation and cell differentiation should be considered. RIG1 stimulates cellular differentiation of keratinocytes, which is mediated by the interaction with and activation of type I tissue transglutaminase [13]. Both RIG1 and H-rev107 interact with prostaglandin D2 synthase in the testes. The interaction enhances prostaglandin D2 synthase activity, which increases prostaglandin D2 levels, activates downstream PGD2 signaling molecules and suppresses cell migration and invasion [25, 46].

Here we demonstrated that POR interacted with H-rev107, enhanced PLA_2_ activity of H-rev107, and increased AA release. The H-rev107 was not the substrate of POR and the amount of H-rev107 protein was not influenced by the presence of POR (Fig 2C).Furthermore, POR did not increase the PLA_2_ activity *in vitro* (Fig 2A), suggesting that the direct interaction between POR and H-rev107 is not involved in the increased AA releasing ability of PLA_2_ activity of H-rev107. AAs are further metabolized to hydroxyeicosatetraenoic acids or epoxyeicosatrienoic acids by the cytochrome P450 pathway [47, 48]. Cytochrome P450 activity is controlled by POR. Future work analyzing the effects of hydroxyeicosatetraenoic acids and epoxyeicosatrienoic acids in cells co-expressing H-rev107 and POR will help us elucidate their roles in AA metabolism via cytochrome P450s pathway.

By contrast, our results show that H-rev107 suppressed POR activity, determined by a reduction in cytochrome c *in vitro* and in cells (Fig 3A and 3C). The expression level of POR was not affected by the presence of H-rev107 (Fig 3C) and both catalytically inactive point mutants of H-rev107 could bind to POR but did not inhibit its activity (S3 Fig and Fig 3D). Furthermore, H-rev107 activity that suppressed POR activity was eliminated when treated with PLA_2_ inhibitors. These results indicate that H-rev107 inhibits POR activity via the PLA_2_ pathway and the binding ability of H-rev107 with POR is independent of POR activity, even a weak inhibition of POR activity was observed when POR co-incubated with higher dose of H-rev107 *in vitro*. Similarly, a catalytically inactive point mutant of H-rev107 devoid of the chaperone activity of Pex19p, was still capable of binding to Pex19p [26].

In addition to inhibiting POR activity, H-rev107 has potential to increase the lipid accumulation in cells. H-rev107 induced AA release which led to an increase in lipid content within cells. However, the AA level within H-rev107 expressing cells was extremely low compared with AA released in culture medium (data not shown). This result suggests that H-rev107 alleviated POR-mediated decreased lipid accumulation in cells via regulation of POR itself. In addition to the cytochrome p450 pathway, AAs are converted to eicosanoids and produce prostaglandin H2 or arachidonic acid 5-hydroperoxide through the prostaglandin-endoperoxide synthase/cyclooxygenase (PTGS/COX) pathway [49] or the lipoxygenase (LOX) pathway [50], respectively. Based on our previous results [46] and this study, we showed that the NC domain of H-rev107 is required for H-rev107 to bind both PTGS and POR, and the H-rev107/PGDS/POR protein complex prefers the PTGS/COX pathway through the activation of prostaglandin D2 synthase and suppresses the cytochrome P450 pathway by inhibiting POR activity. Additionally, the PGE2 levels are drastically decreased in H-rev107 null mice, providing evidence for a significant role for H-rev107 in regulating prostaglandin synthesis [22].

H-rev107-deficient Ob/Ob mice have higher energy expenditure with higher fatty acid oxidation within adipocytes. These results lead to H-rev107 null mice being leaner when fed a high-fat diet than Ob/Ob mice [22]. Reduced PGE2 and increased cAMP have been shown to increases lipolysis in H-rev107 deficient adipocytes. Our results revealed that H-rev107 can affect triglyceride degradation in HeLa derived cells and hepatoma cells. Although cervical and liver cells are not the major known cells to breakdown triglyceride to release glycerol and the cells used in our study may not reflect physiological condition, both HeLa and hepatoma cells had used to examine the effect of HIF-2α or pigment epithelium-derived factor on triglyceride degradation pathway [51, 52]. Whether the H-rev107/POR or the PGE2/cAMP pathway is involved in H-rev107-mediated lipid accumulation in liver cells needs further investigation. Regardless the downstream signal pathway that H-rev107 regulated, H-rev107 knockdown affects triglyceride degradation according to previous [22] and present studies.

Our present results demonstrate for the first time that H-rev107 is associated with POR and inhibits its activity through PLA_2_ activity. Expression of H-rev107 relieved POR-mediated triglyceride content in cells. Silencing of *H-rev107* resulted in increased glycerol production, confirming the role H-rev107 on lipid accumulation in cells. Furthermore, silencing of H-rev107 expression decreases FFA-mediated suppression of cell viability. In addition to phospholipid, we show that H-rev107 is involved in lipid accumulation in cells via the POR pathway.

## Supporting Information

S1 Fig Subcellular localization of H-rev107 and POR. HtTA cells were transiently transfected with H-rev107-myc or GFP-POR expression vector for 18 h. The cells were fixed and then incubated with anti-myc, anti-PDI (ER marker), or anti-GM130 (Golgi marker) antibodies followed by Alexa Fluor^@^ 488 goat anti-mouse IgG and Alexa Fluor^@^ 643 goat anti-rabbit IgG antibodies. The cells were then analyzed with a laser scanning confocal microscope. Scale bar: 10 μm.
(TIF)

S2 Fig H-rev107 co-localizes with POR at the ER apparatus. HtTA cells were transiently transfected with H-rev107-myc or GFP-POR expression vector for 18 h. The cells were fixed and then incubated with anti-myc or anti-PDI antibodies followed by Alexa Fluor^@^ 633 goat anti-mouse IgG and Alexa Fluor^@^ 405 goat anti-rabbit IgG antibodies. The localization of H-rev107 (red), POR (green), and ER (blue) were analyzed using a laser scanning confocal microscope. Arrows indicates the co-localization of H-rev107 and GFP-POR at the ER apparatus. Scale bar: 10 μm.
(TIF)

S3 Fig H-rev107 associates with POR. HtTA cells were transfected with the POR along with the indicated H-rev107 expression or control vector for 24 h. The cell lysates were prepared, and the interaction between H-rev107 and POR was analyzed by immunoprecipitation followed by western blot analysis.
(TIF)

S4 Fig HtTA cells co-expressing H-rev107 and POR. HtTA cells plated in a 6-well plate were transfected with H-rev107-myc and GFP-POR expression vector for 24 h. The cells were fixed and then incubated with anti-myc antibody followed by Alexa fluor 633 anti-mouse IgG antibody. The cells were then analyzed for H-rev107 (red) and POR (green) expression with an immunofluorescent microscope. Scale bar: 50 μm.
(TIF)

S5 Fig Effects of H-rev107 and POR siRNAs on pro-inflammatory cytokine release in Huh7 cells. Huh7 cells were treated with the indicated concentration of FFA for 24 h. The supernatant of cultured Huh7 cells was collected, and the IL-6 (A) and TNF-α (D) release was measured by ELISA. Huh7 cells were transfected with indicated siRNA (B and E) or treated with PLA_2_ inhibitor (C and F) for 24 h and then treated with FFA for 24 h. The culture media were collected and IL-6 (B and C) and TNF-α (E and F) release were measured by ELISA.
(TIF)

## References

[1] Hajnal A, Klemenz R, Schafer R. Subtraction cloning of H-rev107, a gene specifically expressed in H-ras resistant fibroblasts. Oncogene. 1994;9(2):479–490. 8290259

[2] Nazarenko I, Schafer R, Sers C. Mechanisms of the HRSL3 tumor suppressor function in ovarian carcinoma cells. J Cell Sci. 2007;120(8):1393–1404. 17374643 10.1242/jcs.000018

[3] Duncan RE, Sarkadi-Nagy E, Jaworski K, Ahmadian M, Sul HS. Identification and functional characterization of adipose-specific phospholipase A2 (AdPLA). J Biol Chem. 2008;283(37):25428–25436. 10.1074/jbc.M804146200 18614531 PMC2533091

[4] Ren X, Lin J, Jin C, Xia B. 1H, 13C and 15N resonance assignments of human H-REV107 N-terminal domain. Biomol NMR Assign. 2010;4(2):175–178. 10.1007/s12104-010-9238-5 20526701

[5] Huang SL, Shyu RY, Yeh MY, Jiang SY. Cloning and characterization of a novel retinoid-inducible gene 1(RIG1) deriving from human gastric cancer cells. Mol Cell Endocrinol. 2000;159(1–2):15–24. 10687848 10.1016/s0303-7207(99)00207-5

[6] Shyu RY, Hsieh YC, Tsai FM, Wu CC, Jiang SY. Cloning and functional characterization of the HRASLS2 gene. Amino acids. 2008;35(1):129–137. 18163183 10.1007/s00726-007-0612-2

[7] Jin XH, Uyama T, Wang J, Okamoto Y, Tonai T, Ueda N. cDNA cloning and characterization of human and mouse Ca(2+)-independent phosphatidylethanolamine N-acyltransferases. Biochim Biophys Acta. 2009;1791(1):32–38. 10.1016/j.bbalip.2008.09.006 19000777

[8] Ito H, Akiyama H, Shigeno C, Nakamura T. Isolation, characterization, and chromosome mapping of a human A-C1 Ha-Ras suppressor gene (HRASLS). Cytogenet Cell Genet. 2001;93(1–2):36–39. 11474175 10.1159/000056944

[9] Anantharaman V, Aravind L. Evolutionary history, structural features and biochemical diversity of the NlpC/P60 superfamily of enzymes. Genome Biol. 2003;4(2):R11 12620121 10.1186/gb-2003-4-2-r11 PMC151301

[10] Hughes PJ, Stanway G. The 2A proteins of three diverse picornaviruses are related to each other and to the H-rev107 family of proteins involved in the control of cell proliferation. J Gen Virol. 2000;81(1):201–207. 10640559 10.1099/0022-1317-81-1-201

[11] Tsai FM, Shyu RY, Jiang SY. RIG1 inhibits the Ras/mitogen-activated protein kinase pathway by suppressing the activation of Ras. Cell Signal. 2006;18(3):349–358. 16005186 10.1016/j.cellsig.2005.05.005

[12] Tsai FM, Shyu RY, Lin SC, Wu CC, Jiang SY. Induction of apoptosis by the retinoid inducible growth regulator RIG1 depends on the NC motif in HtTA cervical cancer cells. BMC Cell Biol. 2009;10:15 10.1186/1471-2121-10-15 19245694 PMC2656461

[13] Sturniolo MT, Dashti SR, Deucher A, Rorke EA, Broome AM, Chandraratna RA, et al A novel tumor suppressor protein promotes keratinocyte terminal differentiation via activation of type I transglutaminase. J Biol Chem. 2003;278(48):48066–48073. 12928434 10.1074/jbc.M307215200

[14] Scharadin TM, Jiang H, Martin S, Eckert RL. TIG3 interaction at the centrosome alters microtubule distribution and centrosome function. J Cell Sci. 2012;125(11):2604–2614. 22427689 10.1242/jcs.096495 PMC3403232

[15] Scharadin TM, Jiang H, Jans R, Rorke EA, Eckert RL. TIG3 tumor suppressor-dependent organelle redistribution and apoptosis in skin cancer cells. PloS one. 2011;6(8):e23230 10.1371/journal.pone.0023230 21858038 PMC3157364

[16] Tsai FM, Shyu RY, Jiang SY. RIG1 suppresses Ras activation and induces cellular apoptosis at the Golgi apparatus. Cell Signal. 2007;19(5):989–999. 17196792 10.1016/j.cellsig.2006.11.005

[17] Huang SL, Shyu RY, Yeh MY, Jiang SY. The retinoid-inducible gene I: effect on apoptosis and mitogen-activated kinase signal pathways. Anticancer Res. 2002;22(2A):799–804. 12014653

[18] Uyama T, Jin XH, Tsuboi K, Tonai T, Ueda N. Characterization of the human tumor suppressors TIG3 and HRASLS2 as phospholipid-metabolizing enzymes. Biochim Biophys Acta. 2009;1791(12):1114–1124. 10.1016/j.bbalip.2009.07.001 19615464

[19] Uyama T, Morishita J, Jin XH, Okamoto Y, Tsuboi K, Ueda N. The tumor suppressor gene H-Rev107 functions as a novel Ca2+-independent cytosolic phospholipase A1/2 of the thiol hydrolase type. J Lipid Res. 2009;50(4):685–693. 10.1194/jlr.M800453-JLR200 19047760 PMC2656662

[20] Shinohara N, Uyama T, Jin XH, Tsuboi K, Tonai T, Houchi H, et al Enzymological analysis of the tumor suppressor A-C1 reveals a novel group of phospholipid-metabolizing enzymes. J Lipid Res. 2011;52(11):1927–1935. 10.1194/jlr.M015081 21880860 PMC3196224

[21] Uyama T, Ichi I, Kono N, Inoue A, Tsuboi K, Jin XH, et al Regulation of peroxisomal lipid metabolism by catalytic activity of tumor suppressor H-rev107. J Biol Chem. 2012;287(4):2706–2718. 10.1074/jbc.M111.267575 22134920 PMC3268428

[22] Jaworski K, Ahmadian M, Duncan RE, Sarkadi-Nagy E, Varady KA, Hellerstein MK, et al AdPLA ablation increases lipolysis and prevents obesity induced by high-fat feeding or leptin deficiency. Nat Med. 2009;15(2):159–168. 10.1038/nm.1904 19136964 PMC2863116

[23] Deucher A, Nagpal S, Chandraratna RA, Di Sepio D, Robinson NA, Dashti SR, et al The carboxy-terminal hydrophobic domain of TIG3, a class II tumor suppressor protein, is required for appropriate cellular localization and optimal biological activity. Int J Oncol. 2000;17(6):1195–1203. 11078805 10.3892/ijo.17.6.1195

[24] Jans R, Sturniolo MT, Eckert RL. Localization of the TIG3 transglutaminase interaction domain and demonstration that the amino-terminal region is required for TIG3 function as a keratinocyte differentiation regulator. J Invest Dermatol. 2008;128(3):517–529. 17762858 10.1038/sj.jid.5701035

[25] Wu CC, Shyu RY, Wang CH, Tsai TC, Wang LK, Chen ML, et al Involvement of the prostaglandin D2 signal pathway in retinoid-inducible gene 1 (RIG1)-mediated suppression of cell invasion in testis cancer cells. Biochim Biophys Acta. 2012;1823(12):2227–2236. 10.1016/j.bbamcr.2012.08.013 22960220

[26] Uyama T, Kawai K, Kono N, Watanabe M, Tsuboi K, Inoue T, et al Interaction of Phospholipase A/Acyltransferase-3 with Pex19p: A POSSIBLE INVOLVEMENT IN THE DOWN-REGULATION OF PEROXISOMES. J Biol Chem. 2015;290(28):17520–17534. 10.1074/jbc.M114.635433 26018079 PMC4498086

[27] Lu AY, Junk KW, Coon MJ. Resolution of the cytochrome P-450-containing omega-hydroxylation system of liver microsomes into three components. J Biol Chem. 1969;244(13):3714–3721. 4389465

[28] Furge LL, Guengerich FP. Cytochrome P450 enzymes in drug metabolism and chemical toxicology: An introduction. Biochem Mol Biol Educ. 2006;34(2):66–74. 10.1002/bmb.2006.49403402066 21638641

[29] Baer-Dubowska W, Szaefer H. Modulation of carcinogen-metabolizing cytochromes P450 by phytochemicals in humans. Expert Opin Drug Metab 2013;9(8):927–941. 10.1517/17425255.2013.795219 23634851

[30] Waxman DJ. Regulation of liver-specific steroid metabolizing cytochromes P450: cholesterol 7alpha-hydroxylase, bile acid 6beta-hydroxylase, and growth hormone-responsive steroid hormone hydroxylases. J Steroid Biochem Mol Biol. 1992; 43(8):1055–1072. 10.1016/0960-0760(92)90333-E 22217850

[31] Pikuleva I, Waterman M. Cytochromes P450 in synthesis of steroid hormones, bile acids, vitamin D3 and cholesterol. Mol Aspects Med. 1999;20(1–2):33–42, 43–37. 10575650

[32] Shen AL, O'Leary KA, Kasper CB. Association of multiple developmental defects and embryonic lethality with loss of microsomal NADPH-cytochrome P450 oxidoreductase. J Biol Chem. 2002;277(8):6536–6541. 11742006 10.1074/jbc.M111408200

[33] Weng Y, DiRusso CC, Reilly AA, Black PN, Ding X. Hepatic gene expression changes in mouse models with liver-specific deletion or global suppression of the NADPH-cytochrome P450 reductase gene. Mechanistic implications for the regulation of microsomal cytochrome P450 and the fatty liver phenotype. J Biol Chem. 2005;280(36):31686–31698. 16006652 10.1074/jbc.M504447200

[34] Mutch DM, Klocke B, Morrison P, Murray CA, Henderson CJ, Seifert M, et al The disruption of hepatic cytochrome p450 reductase alters mouse lipid metabolism. J. Proteome Res. 2007; 6(10):3976–3984. 17722906 10.1021/pr0700448

[35] Fluck CE, Tajima T, Pandey AV, Arlt W, Okuhara K, Verge CF, et al Mutant P450 oxidoreductase causes disordered steroidogenesis with and without Antley-Bixler syndrome. Nat Genet. 2004;36(3):228–230. 14758361 10.1038/ng1300

[36] Wang CH, Shyu RY, Wu CC, Tsai TC, Wang LK, Chen ML, et al Phospholipase A/Acyltransferase enzyme activity of H-rev107 inhibits the H-RAS signaling pathway. J Biomed Sci. 2014;21:36 10.1186/1423-0127-21-36 24884338 PMC4012743

[37] Makarova O, Kamberov E, Margolis B. Generation of deletion and point mutations with one primer in a single cloning step. BioTechniques. 2000;29(5):970–972. 11084856 10.2144/00295bm08

[38] Guengerich FP, Martin MV, Sohl CD, Cheng Q. Measurement of cytochrome P450 and NADPH-cytochrome P450 reductase. Nat Protoc. 2009;4(9):1245–1251. 10.1038/nprot.2009.121 19661994 PMC3843963

[39] Dennis EA, Cao J, Hsu YH, Magrioti V, Kokotos G. Phospholipase A2 enzymes: physical structure, biological function, disease implication, chemical inhibition, and therapeutic intervention. Chem Rev. 2011;111(10):6130–6185. 10.1021/cr200085w 21910409 PMC3196595

[40] Gelb MH, Jain MK, Berg OG. Inhibition of phospholipase A2. FASEB J. 1994;8(12):916–924. 8088457 10.1096/fasebj.8.12.8088457

[41] Jenkins CM, Mancuso DJ, Yan W, Sims HF, Gibson B, Gross RW. Identification, cloning, expression, and purification of three novel human calcium-independent phospholipase A2 family members possessing triacylglycerol lipase and acylglycerol transacylase activities. J Biol Chem. 2004;279(47):48968–48975. 15364929 10.1074/jbc.M407841200

[42] Porter TD, Banerjee S, Stolarczyk EI, Zou L. Suppression of cytochrome P450 reductase (POR) expression in hepatoma cells replicates the hepatic lipidosis observed in hepatic POR-null mice. Drug Metab Dispos. 2011;39(6):966–973. 10.1124/dmd.111.038562 21368239 PMC3100902

[43] Liu J, Liu Y, Parkinson A, Klaassen CD. Effect of oleanolic acid on hepatic toxicant-activating and detoxifying systems in mice. J Pharmacol Exp Ther. 1995;275(2):768–774. 7473165

[44] Chavez-Tapia NC, Rosso N, Tiribelli C. Effect of intracellular lipid accumulation in a new model of non-alcoholic fatty liver disease. BMC Gastroenterol. 2012;12:20 10.1186/1471-230X-12-20 22380754 PMC3313845

[45] Sers C, Emmenegger U, Husmann K, Bucher K, Andres AC, Schafer R. Growth-inhibitory activity and downregulation of the class II tumor-suppressor gene H-rev107 in tumor cell lines and experimental tumors. J Cell Biol. 1997;136(4):935–944. 9049257 10.1083/jcb.136.4.935 PMC2132501

[46] Shyu RY, Wu CC, Wang CH, Tsai TC, Wang LK, Chen ML, et al H-rev107 regulates prostaglandin D2 synthase-mediated suppression of cellular invasion in testicular cancer cells. J Biomed Sci. 2013;20:30 10.1186/1423-0127-20-30 23687991 PMC3669107

[47] Spector AA. Arachidonic acid cytochrome P450 epoxygenase pathway. J Lipid Res. 2009;50:S52–56. 10.1194/jlr.R800038-JLR200 18952572 PMC2674692

[48] Kroetz DL, Zeldin DC. Cytochrome P450 pathways of arachidonic acid metabolism. Curr Opin Lipidol 2002;13(3):273–283. 12045397 10.1097/00041433-200206000-00007

[49] Smith WL, DeWitt DL, Garavito RM. Cyclooxygenases: structural, cellular, and molecular biology. Annu Rev Biochem. 2000;69:145–182. 10966456 10.1146/annurev.biochem.69.1.145

[50] Clark SR, Guy CJ, Scurr MJ, Taylor PR, Kift-Morgan AP, Hammond VJ, et al Esterified eicosanoids are acutely generated by 5-lipoxygenase in primary human neutrophils and in human and murine infection. Blood. 2011;117(6):2033–2043. 10.1182/blood-2010-04-278887 21177434 PMC3374621

[51] Cao R, Zhao X, Li S, Zhou H, Chen W, Ren L, et al Hypoxia induces dysregulation of lipid metabolism in HepG2 cells via activation of HIF-2alpha. Cell Physiol Biochem. 2014;34(5):1427–1441. 10.1159/000366348 25323790

[52] Dai Z, Zhou T, Li C, Qi W, Mao Y, Lu J, et al Intracellular pigment epithelium-derived factor contributes to triglyceride degradation. Int J Biochem. 2013;45(9):2076–2086. 10.1016/j.biocel.2013.07.008 23886488

