# Supplementary figures and images for: H-rev107 Regulates Cytochrome P450 Reductase Activity and Increases Lipid Accumulation
Source: PLoS One. 2015 Sep 18;10(9):e0138586. doi: 10.1371/journal.pone.0138586 (PMC4575093; doi:10.1371/journal.pone.0138586)

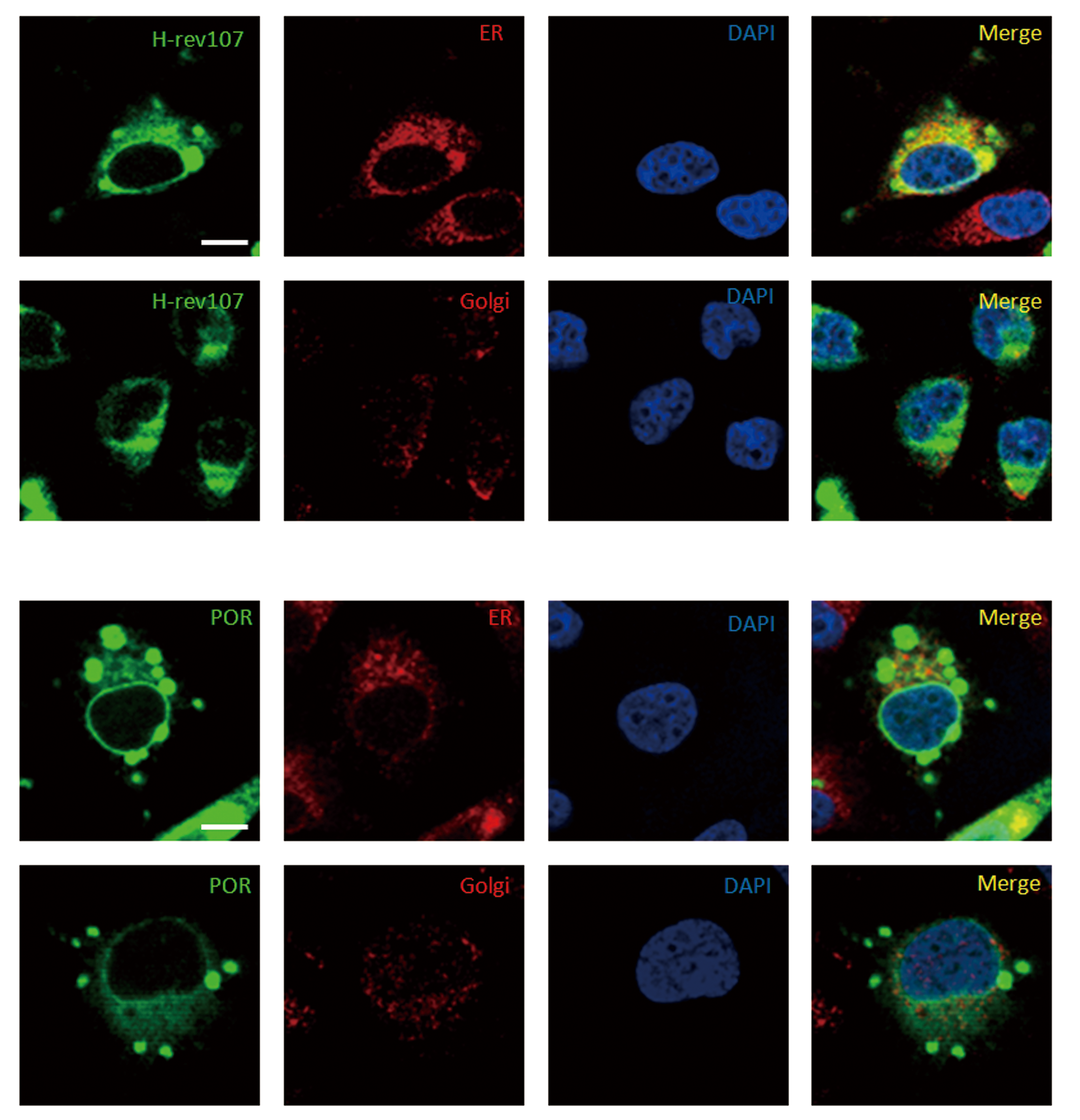

Supplement: S1 Fig — HtTA cells were transiently transfected with H-rev107-myc or GFP-POR expression vector for 18 h. The cells were fixed and then incubated with anti-myc, anti-PDI (ER marker), or anti-GM130 (Golgi marker) antibodies followed by Alexa Fluor@ 488 goat anti-mouse IgG and Alexa Fluor@ 643 goat anti-rabbit IgG antibodies. The cells were then analyzed with a laser scanning confocal microscope. Scale bar: 10 μm. (TIF) [file pone.0138586.s001.tif]

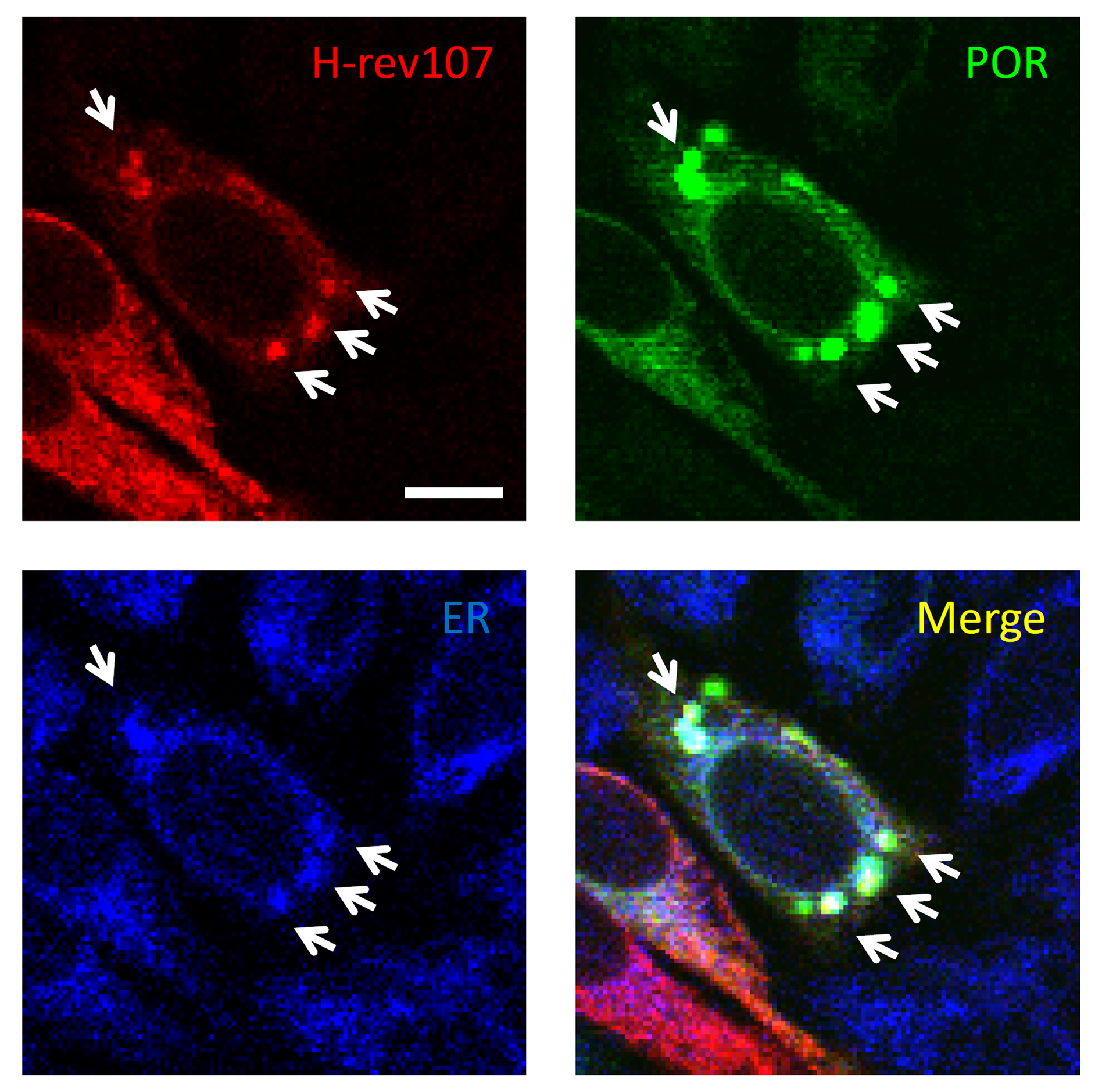

Supplement: S2 Fig — HtTA cells were transiently transfected with H-rev107-myc or GFP-POR expression vector for 18 h. The cells were fixed and then incubated with anti-myc or anti-PDI antibodies followed by Alexa Fluor@ 633 goat anti-mouse IgG and Alexa Fluor@ 405 goat anti-rabbit IgG antibodies. The localization of H-rev107 (red), POR (green), and ER (blue) were analyzed using a laser scanning confocal microscope. Arrows indicates the co-localization of H-rev107 and GFP-POR at the ER apparatus. Scale bar: 10 μm. (TIF) [file pone.0138586.s002.tif]

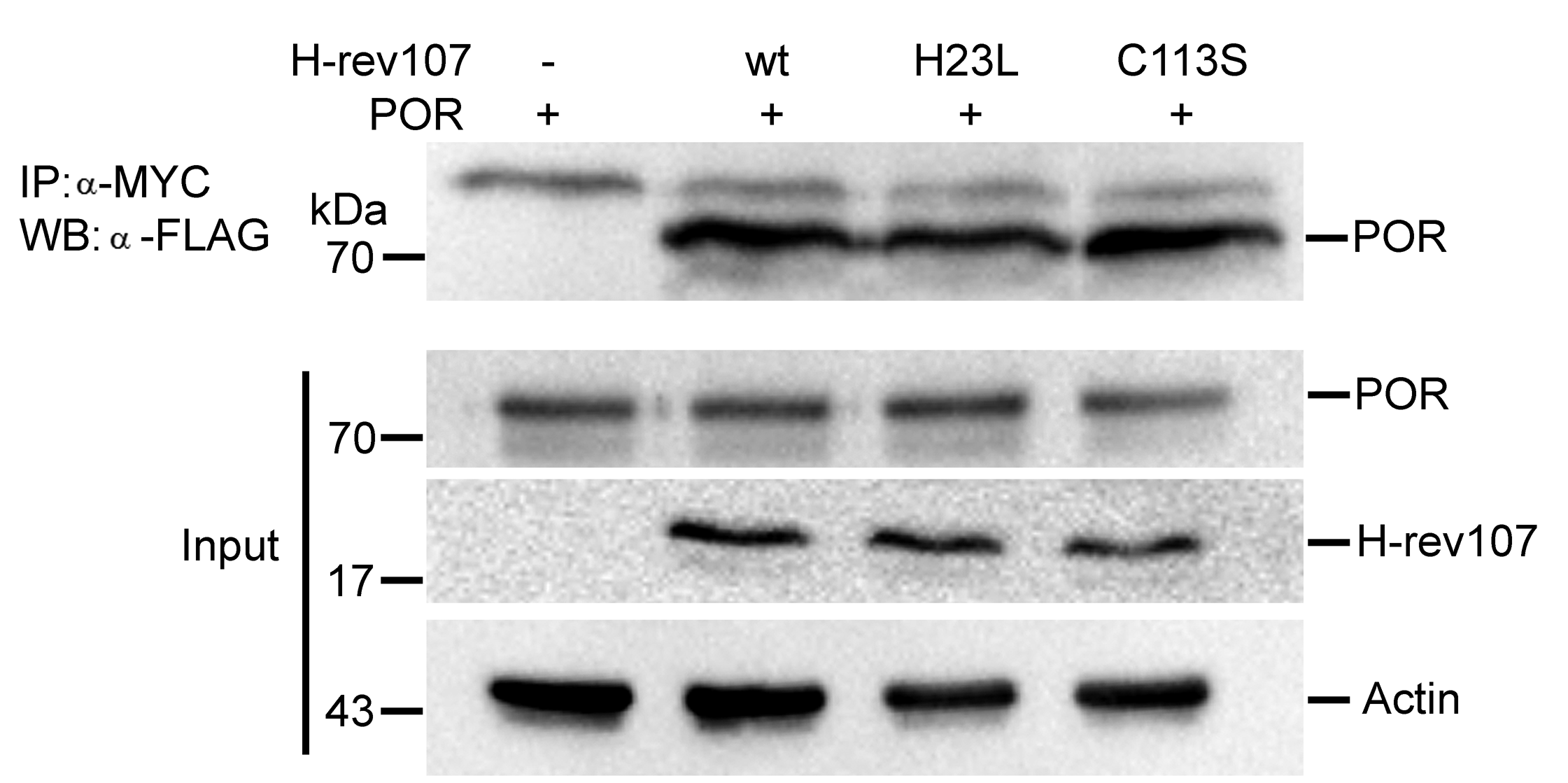

Supplement: S3 Fig — HtTA cells were transfected with the POR along with the indicated H-rev107 expression or control vector for 24 h. The cell lysates were prepared, and the interaction between H-rev107 and POR was analyzed by immunoprecipitation followed by western blot analysis. (TIF) [file pone.0138586.s003.tif]

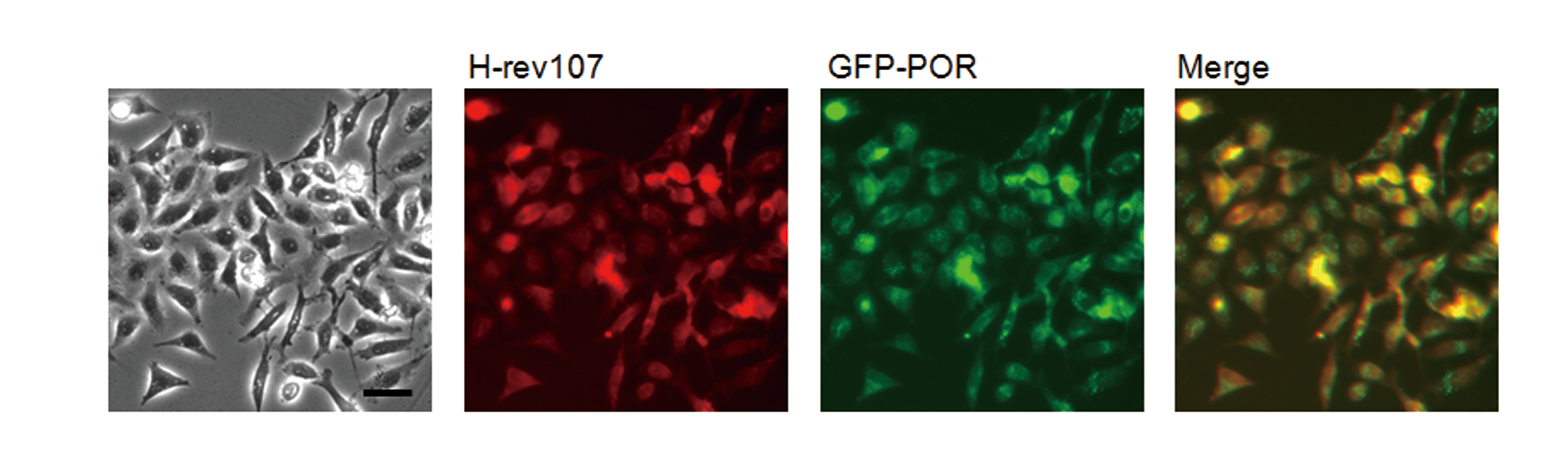

Supplement: S4 Fig — HtTA cells plated in a 6-well plate were transfected with H-rev107-myc and GFP-POR expression vector for 24 h. The cells were fixed and then incubated with anti-myc antibody followed by Alexa fluor 633 anti-mouse IgG antibody. The cells were then analyzed for H-rev107 (red) and POR (green) expression with an immunofluorescent microscope. Scale bar: 50 μm. (TIF) [file pone.0138586.s004.tif]

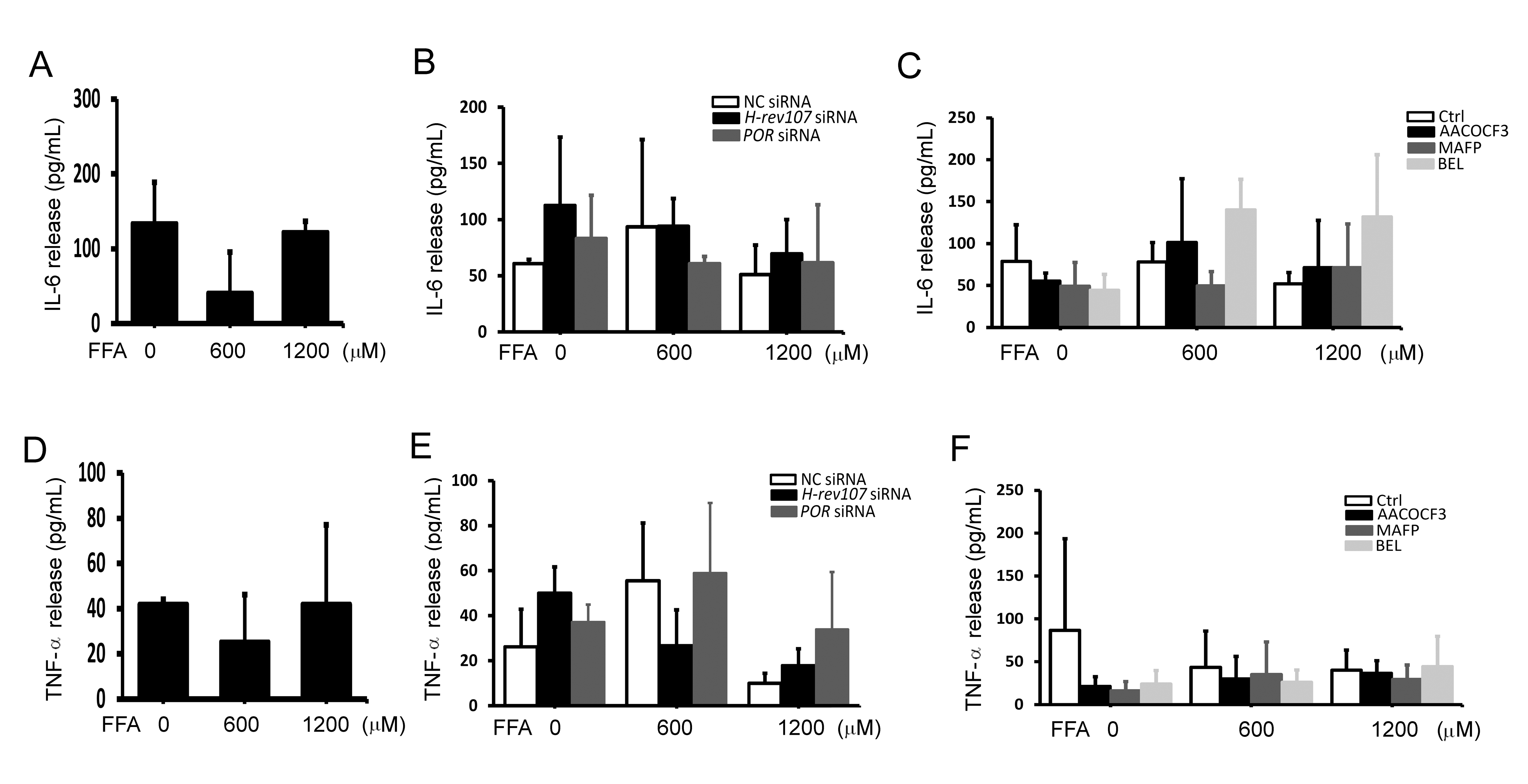

Supplement: S5 Fig — Huh7 cells were treated with the indicated concentration of FFA for 24 h. The supernatant of cultured Huh7 cells was collected, and the IL-6 (A) and TNF-α (D) release was measured by ELISA. Huh7 cells were transfected with indicated siRNA (B and E) or treated with PLA2 inhibitor (C and F) for 24 h and then treated with FFA for 24 h. The culture media were collected and IL-6 (B and C) and TNF-α (E and F) release were measured by ELISA. (TIF) [file pone.0138586.s005.tif]
